# Simulating Directed Evolution *In Silico* with an Iterative, Physics-Based Workflow

**DOI:** 10.1021/acsomega.6c08421

**Published:** 2026-10-11

**Authors:** Chey M. Jones, Deeptak Verma

**Affiliations:** Development Sciences and Clinical Supply, Merck & Co., Inc., 90 East Scott Avenue, Rahway, New Jersey 07065, United States

## Abstract

Directed evolution consistently delivers engineered enzymes that facilitate diverse biocatalytic transformations. Despite advances in automation and high-throughput workflows, our ability to rapidly engineer novel enzymes is limited by our inability to thoroughly sample the vast protein design space. Generative artificial intelligence (AI) methods have alleviated this challenge to some degree, due to their ability to propose mutations based on learned patterns from complex protein data sets. However, the hidden logic of these AI-based methods can complicate our ability to reveal and exploit fundamental details of these enzymes. To complement these hidden-logic models, we present a structure- and physics-based workflow that mimics directed evolution *in silico*. This Virtual Protein EngineeR (referred to as ViPER) aims to simulate directed evolution by leveraging computational modeling, molecular dynamics simulations, *in silico* mutagenesis, and structure-based property evaluation to iteratively engineer an enzyme toward a desired target. In this work, we present a retrospective analysis to demonstrate ViPER’s ability to efficiently navigate the complex protein–protein interfaces associated with the oligomerization of a ketoreductase (KRED) enzyme. In addition to successfully predicting >70% of sites that are crucial for toggling the oligomerization of this KRED enzyme (after only five rounds), this dynamics-based methodology identifies a distal mutation that was incorporated during experiment-based directed evolution. This work provides a novel foundation for computationally driven directed evolution efforts, opening the opportunity to simultaneously pursue and evaluate multiple evolutionary routes through a physics-based lens.

## Graphical abstract

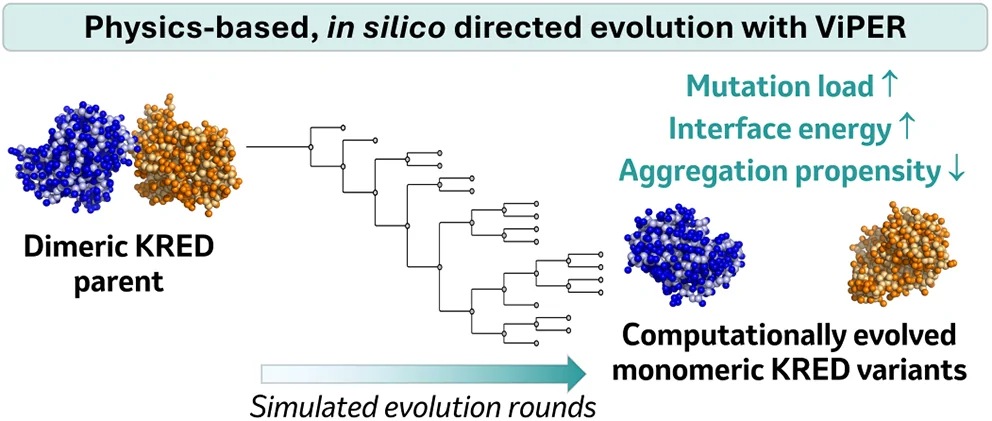

## Introduction

Enzymes are biological powerhouses that enable a diverse range of chemical reactions with exquisite efficiency and selectivity.^1–5^ Directed evolution provides an iterative framework to repurpose naturally evolved enzymes, shifting an enzyme’s catalytic focus from its natural reaction toward a desired reaction.^6–8^ Typically, directed evolution relies on mutating an initial enzyme sequence (*i.e.*, “parent”) to improve its performance under a well-defined selection pressure (*e.g.*, high temperature,^9^ solvent tolerance^10^). Successful directed evolution campaigns generally require multiple iterations of this design and screening approach (*i.e.*, “rounds”), and the result is an engineered enzyme capable of enabling novel and/or more efficient biocatalytic reactions.

The rise of generative artificial intelligence (AI) methods^11–15^ has transformed our ability to efficiently identify promising regions of protein design space during directed evolution.^16–19^ Given a protein sequence or structure, these AI-based methods can leverage learned patterns from hundreds-to-millions of similar sequences to propose mutations for a protein of interest. However, it is unclear how much information about protein dynamics is implicitly embedded in these AI models. Because most proteins are highly dynamic and a small number of mutations can drastically alter a protein’s conformational landscape,^20,21^ AI-suggested mutations based on sequences or static enzyme structures may not account for distal and flexible regions that affect enzyme behavior.^22–24^ Thus, physics-based computational approaches may be necessary for an *a priori* identification of dynamic protein regions that influence enzyme function and properties.

Computational approaches can be developed to understand the physics of complex natural phenomena, as demonstrated for the *in silico* discovery of small molecules.^25–27^ Building on the promise of physics-based approaches for computational enzyme engineering,^28–33^ we present the framework for a Virtual Protein EngineeR (referred to as ViPER) that leverages structure- and physics-based methods to understand and predict enzyme evolution pathways *in silico*. Through an iterative workflow that utilizes structural modeling, coarse-grained molecular dynamics (CGMD) simulations, *in silico* mutagenesis, and a structure-based evaluation of protein properties, ViPER identifies and targets key protein regions and interactions that are revealed on nanosecond-to-microsecond time scales. Rather than relying on machine learning models trained on large data sets, ViPER aims to tune an enzyme’s structural and dynamic properties using information obtained from *in silico* modeling and simulations. This insight allows ViPER to focus on a well-defined subset of protein design space and pursue simultaneous evolution routes *in silico*, enabling computational exploration and engineering to predict promising routes for directed evolution.^34^

Here, we introduce ViPER by simulating the directed evolution of a ketoreductase (KRED) enzyme that was engineered to tolerate high organic solvent conditions for industrial-scale manufacturing.^10^ The high organic solvent tolerance of this KRED enzyme was attributed to the formation of a dimer, making it an intriguing case study to assess ViPER’s physics-informed design strategy at complex protein–protein interfaces. Starting from an engineered KRED dimer (*i.e.*, “KRED-15”), which had evolved from a nearby homolog of a monomeric KRED parent^35^ (*i.e.*, “KRED-1”), our goal for this retrospective study was to evaluate ViPER’s ability to predict mutations that drive the interconversion of these oligomeric states. We demonstrate the physics-based ViPER workflow successfully predicts critical positions at the dimeric protein interface that are hypothesized to impact the oligomerization, activity, and solvent tolerance of this enzyme. Additionally, we observe that accounting for protein dynamics and energetics allows us to discover a non-intuitive, distal mutation (W291Y) that impacts the solvent tolerance of this KRED. The results of this study showcase the ability of a physics-based, computational workflow to explore, evaluate, and propose potential evolutionary routes for engineering complex interfaces of enzymes.

## Results

The *in silico* ViPER workflow aims to reveal and exploit structural dynamics that are essential for enabling protein function.^23,36,37^ Without physics-based simulations, an *a priori* prediction of enzyme regions that form transient protein–protein or protein–ligand interactions is a challenge. Considering dynamic interactions are well-known to facilitate important enzymatic properties (*e.g.*, catalysis,^21,23^ oligomerization,^10,38^ conformational changes^20^), ViPER follows an iterative framework to explore and evaluate potential mutations based on observations from physics-based simulations. Figure 1 illustrates a summary of the ViPER workflow for this KRED enzyme (see Methods for details), which enabled us to mimic directed evolution though the selection of *in silico* KRED (*is*KRED) variants. Over five rounds of *in silico* directed evolution, ViPER enabled us to pursue simultaneous evolution routes that highlighted mutations and positions to prioritize for mutagenesis.

**Figure 1:**
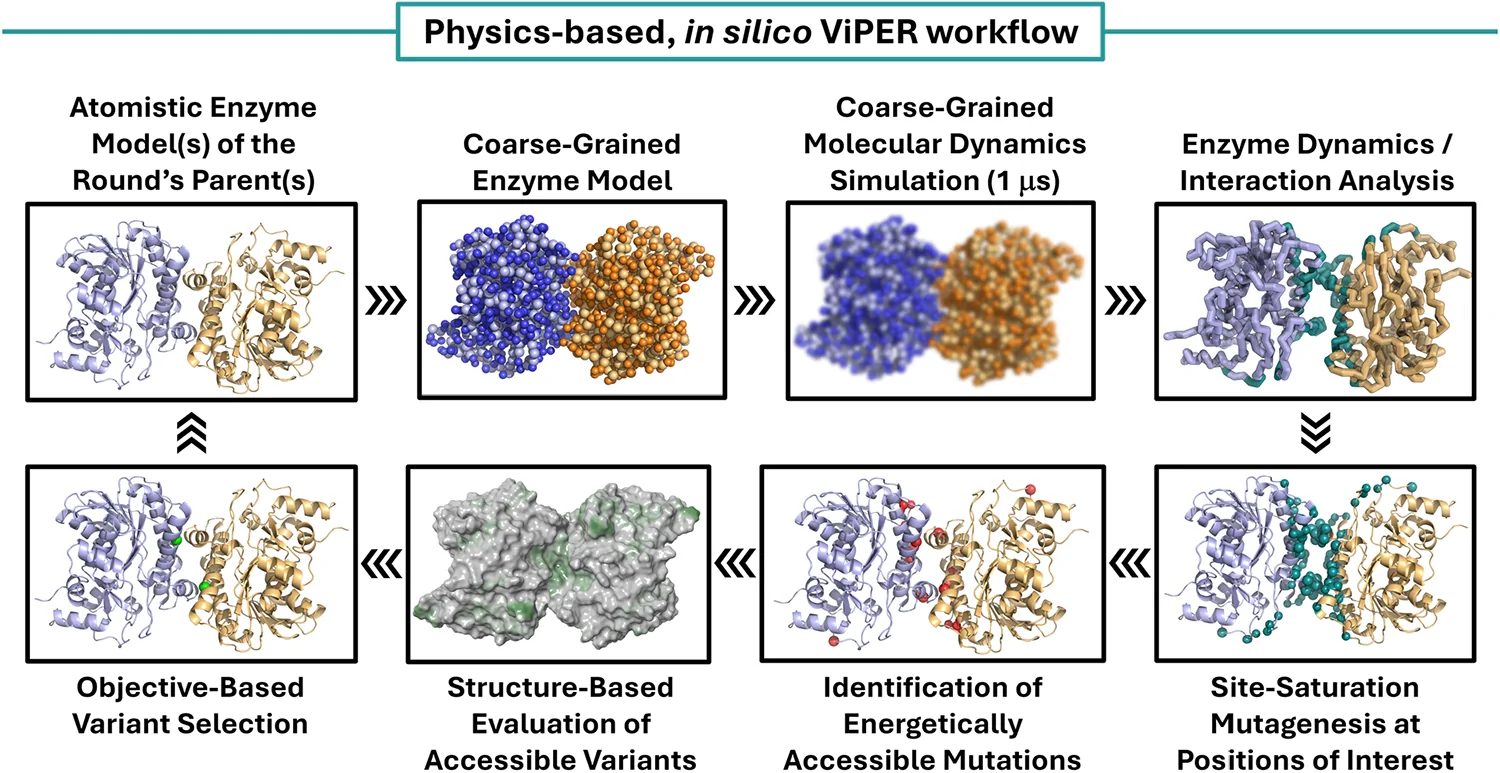
Overview of the physics-based, iterative workflow implemented by ViPER to engineer proteins *in silico*.

### Early-Round Evolution

Starting from an atomistic structural model of the KRED-15 dimer, we generated a coarse-grained structure for the Round 1 parent. This structural coarse-graining groups the atoms of each residue into representative “beads” that simplify the protein structure while retaining the key structural characteristics of the enzyme.^39,40^ Coarse-grained treatment of the solvated KRED-15 system enabled a ∼5× speedup for 1 μs simulations, relative to the respective all-atom system (863 ns/day for coarse-grained versus 182 ns/day for fully atomistic). Because the ViPER workflow relies on information gathered from multiple simulations being performed in parallel, the speedup from CGMD improves the scalability of this dynamics-dependent approach. Leveraging the speedup afforded by coarse-graining, we ran a 1 μs CGMD simulation to identify transient and prolonged protein–protein contacts that occur at the dimeric interface of our initial structure.

With the aim of disrupting interfacial contacts via the introduction of energetically accessible mutations,^38^ the 42 residues that formed dimeric interactions during the CGMD simulation of the Round 1 parent were targeted for site-saturated, *in silico* mutagenesis. Interestingly, only one of these residues −P106—was predicted to have access to mutations that would be well-tolerated based on structural energetics calculated for its monomer and dimer. The crystal structure of the KRED-15 dimer (PDB ID: 9PHG^10^) reveals that P106 is a surface residue within ∼4 Å of the α-helix that houses residues L156–T164 in the adjacent chain (Figure S1). During our 1 μs simulations, we observe favorable interactions between P106 and the V163–T164 residues present in the adjacent chain. Thus, it is plausible that mutating this P106 residue would disrupt these stabilizing interactions and have a significant impact on the oligomerization tendency of this enzyme.

To drive the *in silico* directed evolution toward variants that disrupt the hydrophobic interface that enables dimer formation, we relied on calculated aggregation propensity, *i.e.*, AggScore,^41^ as our selection pressure (Figure 2). AggScore is an *in silico* metric that relies on a protein’s three-dimensional structure to estimate aggregation propensity based on surface characteristics. This structure-based property allowed us to leverage the robust structural data generated for variants each round, providing insight regarding changes to charged and hydrophobic patches in response to specific mutations. Based on the dimeric structures of the 13 P106 variants predicted to be energetically accessible in Round 1, the P106T mutation had the lowest calculated AggScore. Therefore, P106T was chosen as the parent *is*KRED for Round 2 (see Figure 2a). Consistent with experimental findings, the reverse mutation, T106P, played a critical role in engineering a hydrophobic patch that facilitated the monomer-to-dimer transition for this KRED.^10^ ViPER’s ability to correctly identify and incorporate this specific mutation in Round 1 provides evidence that this physics-based approach prioritizes promising regions of protein design space. Furthermore, the presence of the P106T/T106P mutation in multiple directed evolution campaigns—experimental and computational—emphasizes the important role this mutation must play in toggling the KRED’s oligomeric state.

**Figure 2:**
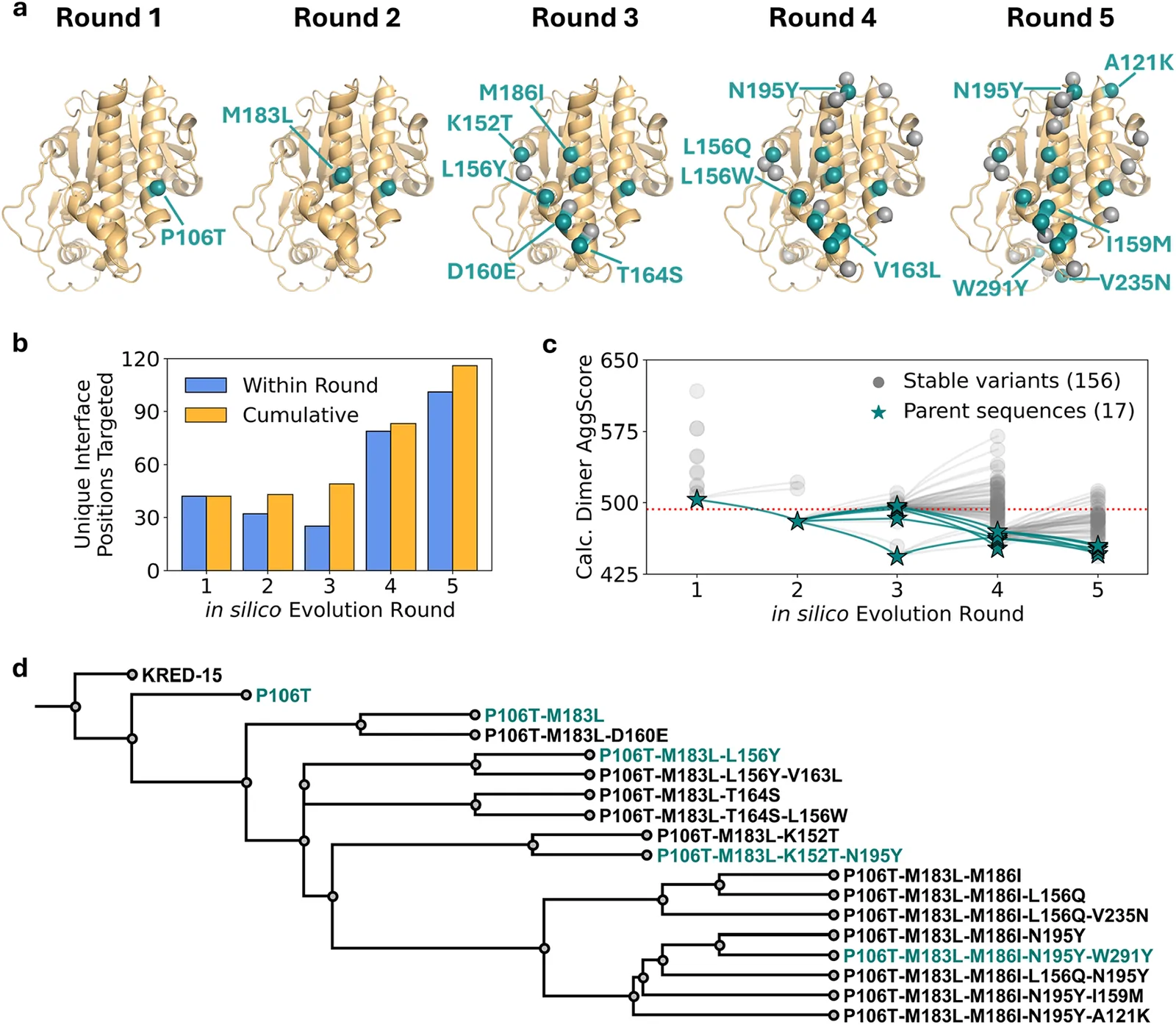
(a) Mutations incorporated during *in silico* directed evolution of a dimeric KRED enzyme. Positions predicted to house energetically accessible mutations are shown as gray spheres (28 positions). Positions modified to yield new parent sequences during evolution are shown as teal spheres (17 variants). For clarity, a monomer unit of the KRED dimer is shown—focusing on the region that forms an interface with the other monomer unit. (b) Number of unique positions targeted for site-saturated mutagenesis during *in silico* evolution. These targeted positions represent residues that formed dimeric contacts during CGMD simulations. The number of unique positions targeted within a given round is represented in blue. The cumulative number of unique positions targeted across rounds is represented in orange. (c) Calculated aggregation score for dimeric structures of variants sampled during *in silico* directed evolution. Aggregation scores for the 156 stable variants (based on Rosetta ΔΔ*G* predictions) discovered during *in silico* evolution are shown in gray. Aggregation scores for the 17 variants chosen as parent sequences during *in silico* evolution are shown in teal. The horizontal red line represents the calculated AggScore for the KRED-15 parent. (d) Phylogenetic tree connecting the *is*KRED parents chosen by ViPER. Variants with the lowest AggScore after each round of *in silico* evolution are highlighted in teal.

As in Round 1 of *in silico* directed evolution, ViPER predicted a single novel site for mutagenesis during Round 2. Figure 2b illustrates that fewer dimeric interactions were observed during the CGMD simulations of Round 2 than in Round 1, likely due to the inclusion of the P106T mutation discovered in the previous round. Of the three energetically accessible mutations predicted for the Round 2 parent (T106A, T106L, and M183L), only one of the proposed choices introduced a mutation at a novel site. In addition to providing a new position to explore in protein design space, the M183L variant had a calculated AggScore that was ∼7% lower (on average) than the other options at position 106 (see Figure 2c). The M183L variant was chosen as the Round 3 parent based on these observations, and a structural analysis suggests this substitution also disrupts contact with the α-helix spanning residues L156–T164 in the adjacent chain (Figure S1). With the incorporation of this M183L mutation into our *is*KRED, ViPER once again discovered a position that seems essential for influencing the dimer formation of KRED-15. We previously reported that a mutation at position 183, *i.e.*, E183M, helped promote the dimerization of KRED-15,^10^ so it is encouraging that ViPER was able to correctly highlight this key position using a physics-driven methodology.

### Mid-Round Evolution

Through two rounds of *in silico* directed evolution with ViPER, only two positions (106 and 183) were classified as promising engineering targets. This resulted in a single parent, P106T–M183L, to begin the third round of evolution. Figure 2b shows that we observed another reduction in the number of contacts formed between the two chains of the Round 3 parent during CGMD simulations. Despite the smaller search space for this round, ViPER predicted 13 mutations (across 10 positions) that were energetically accessible from the Round 3 parent. The corresponding positions were heavily concentrated near the α-helices and loops adjacent to the P106T and M183L substitutions, suggesting these previous mutations afforded the enzyme additional design space in this region. Unlike the first two rounds of evolution, we were able to evaluate multiple potential evolution trajectories in Round 3.

Excluding mutations at any previously modified positions, *i.e.*, positions 106 and 183, stable mutations to the Round 3 parent sequence were evaluated based on the AggScores calculated for their dimeric structures. These mutations were all located near the dimeric KRED interface, as revealed by X-ray crystallography^10^ and structural modeling (Figure 2a). Of the 13 possible stable substitutions to carry forward to Round 4, we selected five *is*KRED variants containing mutations (at unique and previously unvisited positions) that were predicted to have the lowest aggregation propensity—L156Y, T164S, K152T, M186I, and D160E. Three of these five mutations are located at the same α-helix neighboring the P106T and M183L modifications, further emphasizing the role of this interfacial region. Again, we observe that ViPER successfully incorporates a mutation in Round 3—M186I—at a position that constitutes KRED-15’s engineered hydrophobic patch.^10^

### Late-Round Evolution

The presence of multiple, energetically accessible pathways enabled us to simultaneously simulate potential evolution trajectories during the last two rounds of *in silico* directed evolution, an approach that is typically not feasible with physical experiments due to time and resource constraints. Rounds 4 and 5 of *in silico* directed evolution were initiated with five different parent enzymes, resulting in a dramatic increase in the total number of protein–protein interactions observed during these rounds (see Figure 2b). Consequently, ViPER targeted over 100 different positions for mutagenesis (Figure S2), identified over 150 energetically tolerated mutations across 28 positions, and selected *is*KRED parents with AggScores up to 10% lower than KRED-15 by the end of this evolution campaign (Figure 2c).

Unlike the first three rounds, we observe *is*KRED variants dissociate from dimers into monomers in the final two rounds. Figure 3a shows the distribution of minimum distances measured between the *is*KRED dimer subunits during CGMD simulations within each round. The parent structures of Rounds 1 through 3 form sustained interactions over the course of the CGMD simulations. This behavior is reflected by the narrow distance distributions and a maximum distance between the two chains that does not exceed ∼5 Å during these rounds. However, the emergence of mutations that aim to deaggregate this KRED appears to have an impact by Round 4. CGMD simulations suggest that dimer dissociation takes place after hundreds of nanoseconds for the *in silico* P106T–M183L–L156Y and P106T–M183L–D160E KRED variants. Time-resolved distance measurements for these variants are presented in Figure S3.

**Figure 3:**
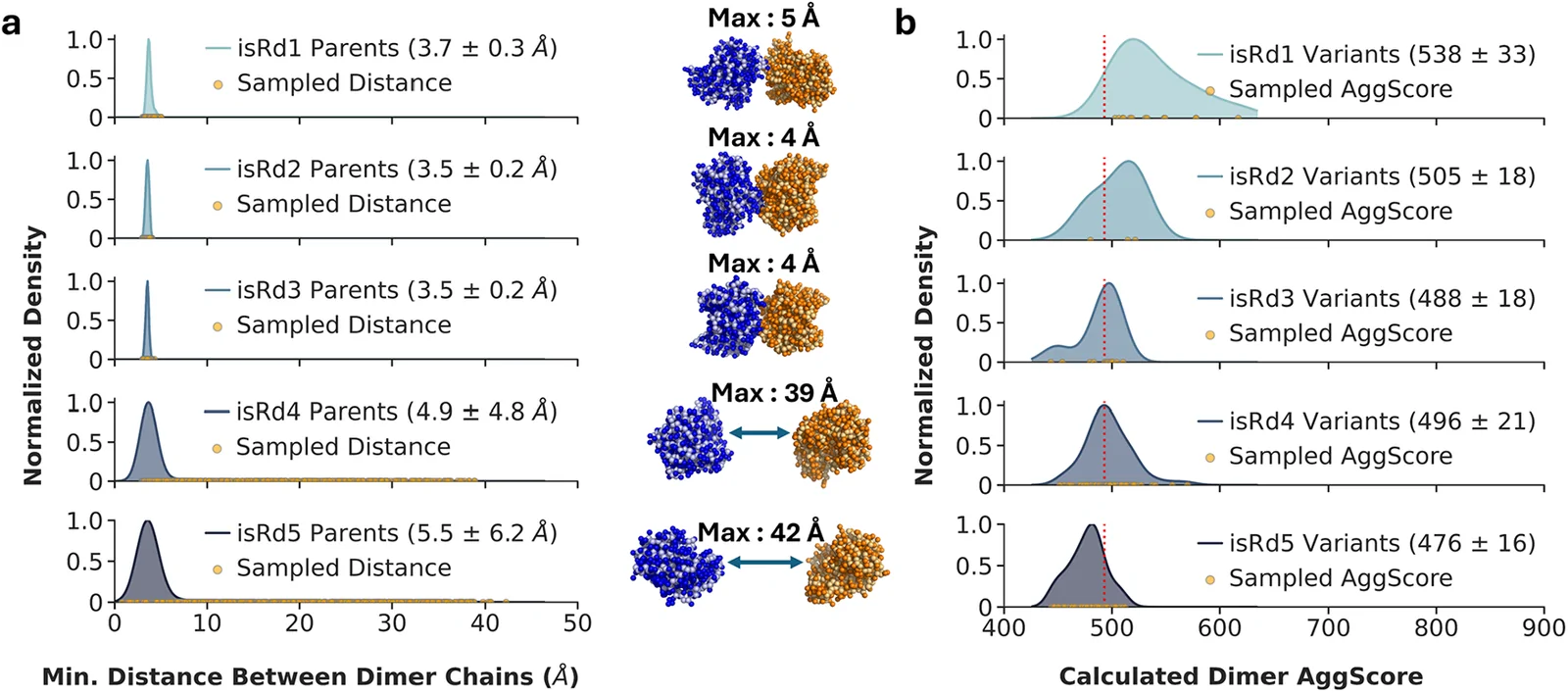
(a) Distribution of the minimum distance between dimer units (*i.e.*, Chain A and Chain B) measured for *is*KRED parents during 1 μs CGMD simulations. Interchain distances calculated for structures sampled during the simulations, within a given round, are shown along the *x*-axis as orange points. The largest separation distance observed within a given *in silico* round (*is*Rd) is illustrated using a representative structure from the CGMD simulation. Chain A is represented as light blue (protein backbone) and dark blue (side chain) beads, whereas Chain B is represented as light orange (protein backbone) and dark orange (side chain) beads. Calculated averages and standard deviations for each data set are shown in parentheses. (b) Distribution of calculated AggScore values for energetically stable *is*KRED variants encountered during *in silico* evolution. Variant structures were predicted with Rosetta. Aggregation scores calculated for dimeric structures of these variants, within a given round, are shown along the *x*-axis as orange points. The vertical red line represents the calculated AggScore for the parent KRED-15. Gaussian kernel density estimation was used to represent the density of measured distances and aggregation scores within a round, and the distributions were normalized such that the maximum y-value was equal to one.

Although dimer dissociation appears to be an uncommon event for the Round 4 variants (see Figure S3), measured separation distances of ∼39 Å indicate that the interface mutations incorporated by some ViPER variants successfully disrupt the aggregation propensity at the dimeric interface. This is reflected by the gradual shift in the calculated dimer AggScore distribution throughout the evolution (Figure 3b). Based on their potential to suppress KRED dimerization, the following five variants were selected as parents for Round 5: (1) P106T–M183L–L156Y–V163L, (2) P106T–M183L–T164S–L156W, (3) P106T–M183L–K152T–N195Y, (4) P106T–M183L–M186I–L156Q, and (5) P106T–M183L–M186I–N195Y. Notably, ViPER captured a substitution at position 163 in this round, which is another member of the engineered dimeric interface for KRED-15.^10^ Two selected variants contained mutations to N195, which is a solvent-exposed residue that forms transient interactions with the neighboring chain. Similarly, modifications to residue L156 were pursued by multiple parents in parallel, likely due to the residue’s proximity to H190 in the adjacent chain (Figure S4).

Round 5 proceeded in a similar fashion to that of Round 4. Starting from five parent enzymes, parallel CGMD simulations were run to identify potential contacts of interest within their respective evolutionary trajectories. Again, we observed two parents, P106T–M183L–M186I–N195Y and P106T–M183L–M186I–L156Q, that experienced significant dimer separation, *i.e.*, greater than 35 Å, during their simulations (Figure S3). Prior to separation, we generally observe transient interactions between residues not located at the primary dimer interface (Figure S5). As a result, Round 5 led to the emergence of mutations at two distal sites – W291Y and V235N (Figure 2a). When introduced to their respective parent sequences, these distal mutations decreased the predicted aggregation propensity of the *is*KRED dimer (see Tables S1–S5). As with the P106T mutation, ViPER captured another specific mutation observed during the engineering of KRED-15 from KRED-1. We hypothesized that the Y291W surface mutation improved KRED loading when engineering from a monomeric KRED toward a dimeric KRED.^10^ Considering our *in silico* evolution occurred in the reverse direction (*i.e.*, from dimeric KRED toward monomeric KRED), the identification of the reverse mutation, W291Y, at a distal, surface site demonstrates the predictive capabilities of the physics-based framework afforded by ViPER.

A summary of the final five variants selected at the end of Round 5 is provided in Table 1. The P106T–M183L–M186I–N195Y–W291Y *is*KRED variant was predicted to have the lowest aggregation propensity at the end of Round 5 (Table S5). Four of the five positions modified in this proposed design, *i.e.*, positions 106, 183, 186, and 291, overlap with positions modified during experiment-based directed evolution.^10^ Furthermore, we observe a strong overlap between the KRED positions modified *in silico* and the positions modified during experiment (see Table 1). This behavior indicates that ViPER efficiently pursues routes that optimize a desired objective, enabling this methodology to guide experiments based on a computational screening of potential evolutionary routes.

**Table 1:** Summary of *is*KRED Variants Selected at the End of Each Round of *In Silico* Directed Evolution (*is*Round) with ViPER^a^ ^,^ ^b^

	*is*Round 1	*is*Round 2	*is*Round 3	*is*Round 4	*is*Round 5
Selected Variant #1	P106T^†^	P106T^†^–M183L^†^	P106T^†^–M183L^†^–M186I^†^	P106T^†^–M183L^†^–M186I^†^–N195Y	P106T^†^–M183L^†^–M186I^†^–N195Y–W291Y^†^
Selected Variant #2	–	–	P106T^†^–M183L^†^–T164S	P106T^†^–M183L^†^–M186I^†^–L156Q	P106T^†^–M183L^†^–M186I^†^–N195Y–I159M
Selected Variant #3	–	–	P106T^†^–M183L^†^–D160E	P106T^†^–M183L^†^–T164S–L156W	P106T^†^–M183L^†^–M186I^†^–N195Y–A121K
Selected Variant #4	–	–	P106T^†^–M183L^†^–L156Y	P106T^†^–M183L^†^–L156Y–V163L^†^	P106T^†^–M183L^†^–M186I^†^–L156Q–V235N
Selected Variant #5	–	–	P106T^†^–M183L^†^–K152T	P106T^†^–M183L^†^–K152T–N195Y	P106T^†^–M183L^†^–M186I^†^–L156Q–N195Y

a The † superscripts indicate that mutation occurs at a position modified during experiment-based directed evolution.

b Variants were selected at the end of each round based on energetic accessibility and their ability to minimize aggregation propensity of the KRED dimer. These *in silico* mutations were compared to mutations accumulated during experiment-based directed evolution.^10^ However, this table has not been previously published and presents an original analysis for the evaluation of ViPER.

### Modification of Structural Interactions and Properties

Although the AggScore calculated during our *in silico* evolution campaign provides a useful approximation for predicting oligomerization potential, protein oligomerization is ultimately determined by the interactions that occur at the enzyme interface. As previously discussed, the protein–protein interactions at the dimeric interface have a major impact on the aggregation propensity of this KRED.^10^ With the intent of identifying the KRED-15 interactions that most likely facilitate enzyme dimerization, we ran 200 ns of all-atom molecular dynamics simulations (AAMD) on the KRED-15 dimer. Figure 4a provides a set of interchain contacts that are retained by the end of these AAMD simulations for KRED-15, and our hypothesis was that these would be the major sites targeted by ViPER to disrupt protein–protein binding. Upon comparison to the set of positions flagged by our CGMD simulations (see Figure S2), we observe that ViPER explored mutations at every residue in Figure 4a that forms prolonged contacts in our AAMD simulation. Of the 19 contact pairs identified for KRED-15, 11 pairs were disrupted during experiment-based directed evolution.^10^ Variants selected during our *in silico* directed evolution protocol aimed to disrupt 17 of these 19 contact pairs, indicating ViPER’s emphasis on driving this KRED toward a monomer (see Figure 4b).

**Figure 4:**
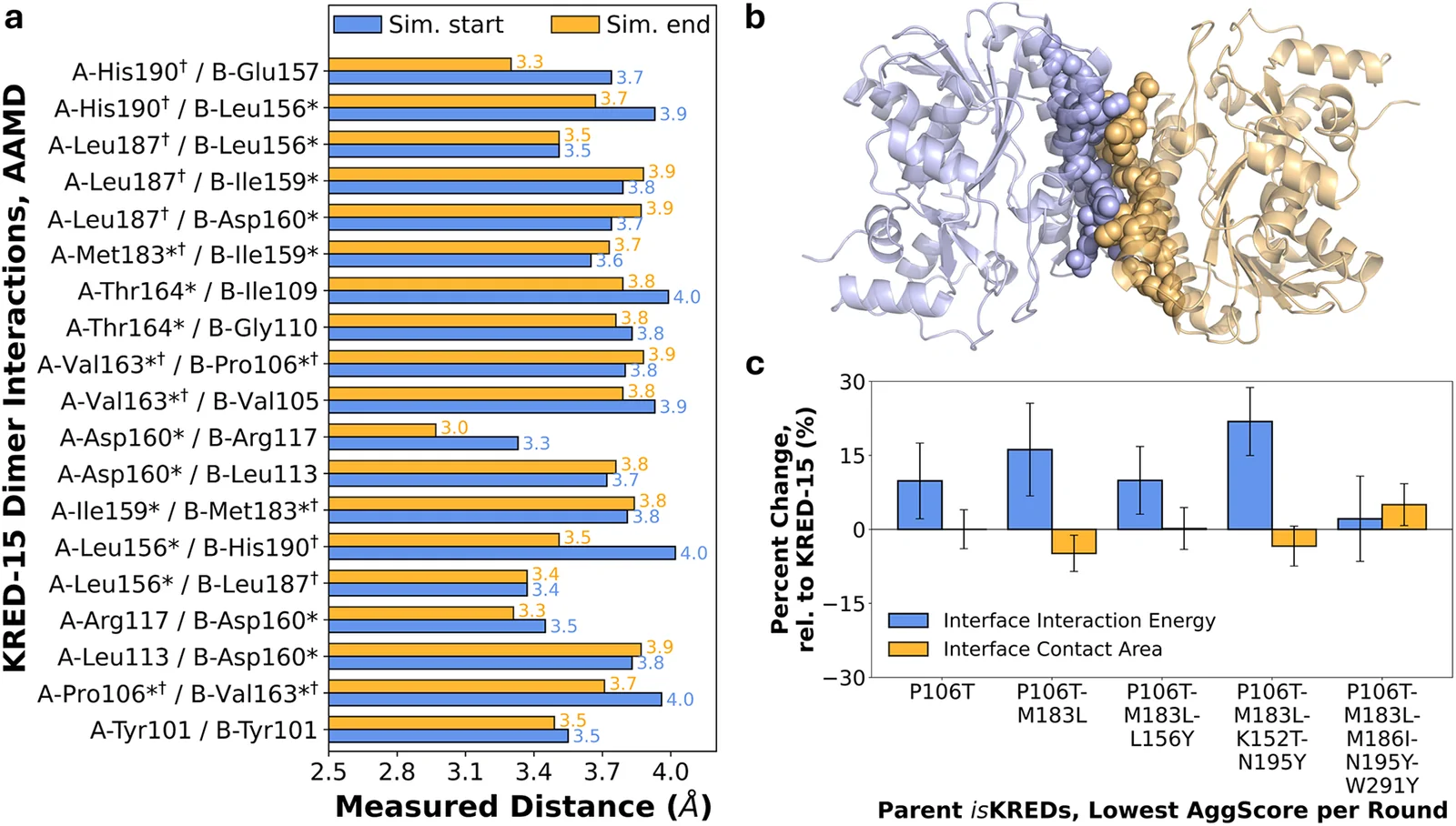
(a) Maintained interactions at the KRED-15 dimer interface after 200 ns of AAMD simulations. Protein contacts (within 4 Å) maintained after 200 ns were identified by measuring structural distances at the start (blue) and end (orange) of the 200 ns AAMD simulation. Residues denoted with an asterisk (*) represent residues that were modified during *in silico* evolution. Residues denoted with a dagger (†) represent residues that were modified during experiment-based directed evolution. (b) Structural overview of KRED-15 residues involved in maintained interactions (spheres) during 200 ns of AAMD simulations. (c) Comparison of dimer interface properties for *in silico* KRED parents, relative to the KRED-15 enzyme. Structures obtained during the final 100 ns of 200 ns AAMD simulations (21 evenly spaced snapshots) were used for this analysis. Percent changes for each variant are calculated relative to the average interface interaction energy and contact area calculated for KRED-15 over the last 100 ns of AAMD. Standard deviations are represented by error bars.

Figure 4a also highlights the complementarity of the experimental and *in silico* directed evolution routes. While we do not detect a complete overlap of modified residues when comparing both engineering approaches, we find that these two methods may be targeting the same interactions, albeit at different residues. For example, ViPER incorporated a mutation at L156 in multiple evolutionary pathways (see Table 1). There were no mutations to position 156 incorporated during the original evolution of this enzyme.^10^ However, the experiment-based engineering campaign of KRED-15 did adopt mutations at positions 187 and 190—two positions predicted to form sustained interactions with L156 but not modified *in silico* (Figure 4a). Thus, it appears that ViPER offers alternative pathways to modify key regions of design space that can be used to complement traditional methods.

To investigate the impact of accumulated mutations on the KRED interface, we ran AAMD simulations to calculate average interface interaction energies and interface contact areas for the top variant selected after each round of *in silico* evolution. Figure 4c illustrates that parent *is*KRED variants selected by ViPER generally increase the interaction energy at the dimer interface by ∼5–20% (relative to the starting KRED-15 enzyme). This observation aligns with the expected behavior of disrupting the interface via mutagenesis. For reference, we modeled a dimeric structure of KRED-1 and ran an AAMD simulation to estimate its interface interaction energy. Consistent with KRED-1’s propensity to exist as a monomer, the average interface interaction energy of KRED-1 is ∼67% greater than that of KRED-15 (Table S6). Other than the Round 2 parent, we did not observe a significant decrease in the interaction contact area (*i.e.*, outside of standard deviation) by introducing these various mutations. This may be a consequence of the shorter time scales of these AAMD simulations (200 ns) versus the longer time scales of CGMD simulations (1 μs), where dissociation behavior was only observed after hundreds of nanoseconds (see Figure S3). However, we did observe a decrease of ∼5% in the interface contact area with the P106T–M183L variant, which may have afforded the *is*KRED with the additional mutational space to explore during Round 3.

### Evolutionary Space Prioritized by ViPER

After five rounds of *in silico* directed evolution, ViPER identified 28 positions that should be prioritized for engineering based on energetic accessibility. Figure 5a illustrates the various positions that were energetically accessible during each round of the *is*KRED evolution in this study. Because these positions are predicted to be engineerable sites, ViPER categorizes them as high-priority residues for design purposes. Although not all 28 of these positions were modified by ViPER during Rounds 1 through 5, building small libraries that modify these high-priority sites could enable prospective designs for any future engineering efforts. Figure 5b highlights sites prioritized by ViPER for KRED-15. Upon comparison to the sites modified during the experiment-based evolution for this KRED,^10^ it is clear that most of the residues prioritized by ViPER are concentrated at the enzyme’s dimeric interface. Considering our computational selection pressure was heavily focused on optimizing properties near the enzyme interface, it is unsurprising that the residues prioritized *in silico* represent a minor subset of the positions modified during the KRED-1 to KRED-15 engineering campaign.

**Figure 5:**
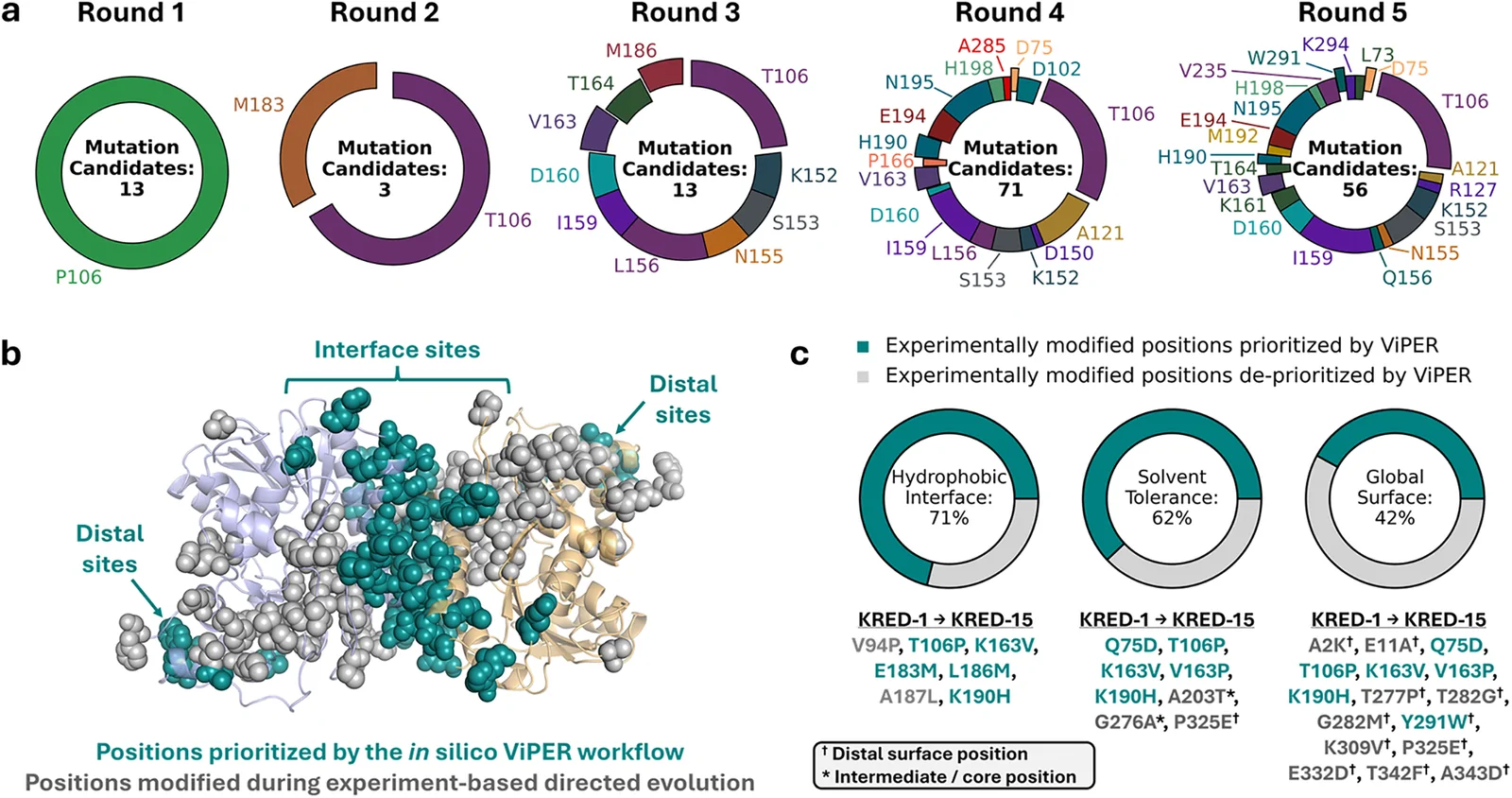
(a) High-priority positions, based on energetic accessibility, identified by ViPER across five rounds of *in silico* directed evolution. The number of mutation candidates represents the number of energetically accessible mutations available to the parent *is*KRED enzymes during that round. Wedges that extend from the donut chart represent positions that were modified during experiment-based evolution to yield KRED-15 from KRED-1. (b) Location of positions prioritized by ViPER (teal spheres) and locations of positions modified during the experiment-based directed evolution (gray spheres). (c) Fraction of key KRED positions, which enable the interchange of KRED-1 and KRED-15, that were successfully categorized as high-priority positions by ViPER during *in silico* directed evolution. Mutations were grouped into functional subsets to evaluate ViPER’s ability to identify positions engineered to form the hydrophobic interface (left), improve solvent tolerance (middle), or redesign the protein surface (right). Positions were categorized based on conclusions from experiment-based directed evolution.^10^ However, this figure has not been previously published and presents an original analysis for the evaluation of ViPER.

Figure 5c highlights the overlap we observed between the positions prioritized by ViPER and the mutations that enabled the dimerization and solvent tolerance of KRED-15.^10^ Seven mutations (*i.e.*, V94P, T106P, K163V, E183M, L186M, A187L, and K190H) were attributed to the emergence of KRED-15’s hydrophobic patch,^10^ and ViPER categorized five of those positions as high-priority sites within the first five rounds of *in silico* directed evolution. While operating in a more focused design space than typical generative AI methods, the ViPER workflow outperformed multiple, zero-shot prediction methods based on their ability to identify these engineerable patches at the interface (see Table S7). Furthermore, it appears that ViPER indirectly identified an additional position at the hydrophobic interface that promotes dimer formation. Although position 187 was not classified as a high-priority mutation, the *is*KRED adopted substitutions at three positions (*i.e.*, positions 156, 159, and 160) predicted to form prolonged interactions with that site (see Figure 4a).

Because dimer formation likely enabled the high organic solvent tolerance of this KRED,^10^ it is plausible that the majority of mutations that toggle aqueous and organic solvent tolerance should be concentrated near the KRED-15 dimer interface. Eight mutations were speculated to improve the KRED’s tolerance toward conditions with high organic solvent ratios. Five of these mutations (62%) fall within ViPER’s high-priority designation. Excluding buried (*i.e.*, located in the intermediate or core regions) and distal positions, ViPER successfully identified all positions near the interface that impacted the enzyme’s solvent tolerance during evolution (Figure S6). Considering the ViPER search space was heavily biased toward the enzyme interface, it is unsurprising that the majority of distal mutations were not identified based on our selection pressure. The only distal mutation identified during this study was the aforementioned W291Y mutation, which was adopted during Round 5 of *in silico* directed evolution. Our emphasis on interfacial modifications also explains the lower capture rate (42%) of surface mutations reported between the KRED-1 and KRED-15 sequences, relative to the capture rate of the residues that constitute the hydrophobic patch (71%). However, it is reassuring that the physics-based nature of ViPER successfully revealed most of the key positions in our region of interest, along with a distal W291Y mutation that would not have been identified by only targeting interface residues suggested by the static structure (see Tables S8–S9).

## Discussion

The *in silico* ViPER workflow presented here seeks to guide the engineering of complex protein–protein interfaces, which is a common challenge during directed evolution. To evaluate ViPER’s ability to highlight key regions for focusing potential design efforts, we simulated directed evolution on a dimeric KRED-15 enzyme that originated from a monomeric KRED variant.^10^ Considering the dimeric nature of the KRED-15 enzyme likely imposes strict constraints on the available protein design space,^42^ our goal was to apply a physics-based methodology to identify key protein–protein interactions and discover mutable enzyme regions available for toggling this KRED’s oligomerization state.

During our *in silico* directed evolution campaign, we observe that our iterative, computational methodology successfully captures many of the positions known to drive this KRED’s interconversion between a monomer and dimer. Previous enzyme engineering efforts have reported that a few modifications at an oligomeric interface can drastically alter a protein’s oligomerization state,^43,44^ and the recurring proposal of mutations at position 106 by ViPER suggests that this position plays a key role in modulating the interfacial character of this KRED. After only five rounds, simulated directed evolution selected *is*KRED parents that modified the majority of positions known to form/disrupt the interfacial hydrophobic patch of this enzyme (*i.e.*, mutations at P106, V163, M183 and M186). Additionally, ViPER categorized 28 positions as high priority for mutagenesis, based on the interactions formed during molecular dynamics (MD) simulations and the energetic accessibility of their mutations. This class of promising positions supplements mutations adopted during *in silico* evolution, and these high priority positions include sites hypothesized to impact the hydrophobic interface (*e.g.*, H190), the solvent tolerance of the enzyme (*e.g.*, D75) and KRED loading (*e.g.*, W291).^10^

Our results establish a proof-of-concept for utilizing an iterative, computational workflow to inform and guide protein engineering efforts. Unlike opaque AI-based methods, this physics-based framework allows us to identify, understand, and modify interactions that influence enzyme behavior. AI-based methods generally aggregate mutations that are likely to be tolerated by an enzyme based on learned patterns within a protein family.^11–13,45^ However, these AI-suggested mutations are usually distributed across the enzyme and can have a broad range of impacts on protein behavior. The dynamics-based approach implemented by ViPER provides a design methodology that aims to highlight engineerable residues that can manipulate *specific* properties of an enzyme, which provides a pathway for efficiently designing small libraries with well-defined objectives. With an *a priori* knowledge of the hydrophobic patch engineered for this KRED enzyme, we selected the calculated AggScore as our *in silico* scoring metric to guide variant selection. This selection allowed us to concentrate our engineering efforts on sites likely to promote or disrupt oligomerization. For prospective implementations of this ViPER methodology, it is critical to ensure that the *in silico* selection pressure is modified to drive the desired changes in the protein properties of interest.

The stochastic, dynamics-based nature of ViPER enables diverse design generation, due to its ability to simulate and evaluate multiple evolutionary pathways for a given enzyme. Using well-defined selection pressures that align with experimental objectives, we can predict key positions and/or mutations responsible for certain protein features. With the purpose of biasing the oligomerization tendencies of an enzyme, we focused this study on correctly pinpointing the most promising design routes available at a KRED’s protein–protein interface. The ViPER methodology establishes a computational framework that can be expanded to optimize various protein properties of interest. For example, environment-specific stability of an enzyme can be predicted by targeting residues with high solvent exposure during CGMD simulations (instead of the contact-based targeting criteria used in this work), and the *in silico* selection pressure can be modified to capture the predicted stability impact of sampled mutations (instead of the AggScore pressure used in this work). Additionally, we can couple this workflow with cofolding methods (e.g., Boltz^46,47^ ) to account for the potential impact of mutations on protein folding. For future efforts, we plan to expand this workflow to investigate ViPER’s ability to proactively design libraries that improve other protein properties (*e.g.*, thermostability) and protein–ligand interactions, which would enable proteins to accommodate non-natural substrates.

## Methods

### Atomistic Enzyme Modeling

The KRED-15 dimer (PDB ID: 9PHG^10^) was used as the structural template to generate all dimeric structures for parent enzymes accumulated during *in silico* directed evolution. Homology models for the parent enzymes were generated using Schrödinger’s Maestro suite and Protein Preparation Workflow.^48–50^ The corresponding structures were refined via a restrained minimization with the OPLS4 force field^51^ (maximum root-mean-square deviation of 0.30 Å). Hydrogens were added to the structures using PROPKA^52^ (pH = 7.4). Monomeric forms of each atomistic structure were generated by removing one of the chains from the dimeric model.

### Coarse-Grained Modeling

The martinize2 protocol^40,53,54^ was used to convert atomistic structures of parent enzymes into coarse-grained representations. Coarse-grained structures were generated using an elastic network model (see Supporting Information for benchmarking details). The insane protocol^55^ was used to build a cubic box of coarse-grained water and Na^+^/Cl^–^ counterions that solvates the coarse-grained structures. Dimensions of 15 nm × 15 nm × 15 nm were used to define the box, with a salt concentration of 0.15. The Martini3 force field^40,56–60^ was used to define parameters for the coarse-grained beads that represent standard amino acid residues, water molecules, and Na^+^/Cl^–^ counterions.

### Coarse-Grained Molecular Dynamics Simulations and Analysis

MD simulations of the coarse-grained systems were conducted using GROMACS.^61–66^ A short energy minimization (10 ps simulation, 1 fs time step) was run to resolve any steric clashes and eliminate any high forces that existed between the beads. A position-restrained NPT equilibration (2 ns) was run to find a local minimum for the system, gradually increasing the simulation time step (10 fs). After equilibrating the system, production-scale MD simulations were run for 1 μs using a time step of 20 fs. The resulting trajectories were analyzed using standard GROMACS functions (*e.g.*, trjconv, rms, rmsf, sasa) and the mdtraj Python package.^54^ Contact pairs at the KRED dimer interface were identified by monitoring which residues (belonging to different chains) came within 4 Å of each other during the 1 μs coarse-grained simulation. To validate the parameters used to define our coarse-grained systems, root-mean-square deviations were calculated for individual protein chains during CGMD simulations and compared to their all-atom counterparts (Figure S7). Statistics related to the sampling of interfacial contacts across CGMD simulations are provided in Table S10.

### *In Silico* Site-Saturated Mutagenesis

Once positions of interest were identified during the coarse-grained simulations, we returned to the atomistic model of the parent enzyme to facilitate *in silico* site-saturated mutagenesis. At each position of interest, we used the Rosetta^67–69^ fixed backbone design (*i.e.*, fixbb) and structure relaxation (*i.e.*, relax) applications to generate all possible single-mutation variants. Single mutations were introduced to both the monomeric and dimeric forms of the enzyme. Based on the structures obtained from Rosetta, the stability of each atomistic, site-saturated mutagenesis variant was evaluated relative to the energy calculated for the respective round’s parent enzyme. Synonymous mutations (*e.g.*, H190H) were used as replicates to calculate stability statistics for the parent enzyme. With the intent of guiding a smooth dimer-to-monomer transition, we sought to ensure that each mutation selected during our *in silico* evolution had a reasonable likelihood of being tolerated by both oligomeric forms. We hypothesized that this approach would allow us to consistently target modifiable sites while reducing the risk of suggesting mutations that inactivate the enzyme. Any single mutation with a monomer *and* dimer Δ*G* lower than the respective parent’s Δ*G* (and outside of the standard deviation calculated from the parent’s statistics) was considered a stable mutation. All stable mutations were considered as candidates for the round’s best variant.

### Structure-Based Variant Evaluation and Selection

Rosetta-generated structures (monomers and dimers) associated with each stabilizing mutation were compiled and analyzed to evaluate each round’s most promising variants.^70,71^ Because the goal of our *in silico* evolution was to find mutations that monomerize the initial KRED dimer, we focused on identifying variants that introduced mutations that minimize the aggregation propensity at regions that form dimeric interactions. To quantify this aggregation propensity, we relied on the structure-based AggScore^41^ calculated using Schrödinger’s protein descriptors. Thus, the AggScore was calculated for all monomeric and dimeric structures associated with stabilizing mutations. Based on the objective of minimizing the AggScore of the dimer near the interface, stabilizing variants with the lowest predicted dimeric AggScore were chosen as the best mutations to move forward to the next round of *in silico* evolution (see Tables S1–S5 for monomer data). With the goal of pursuing multiple evolutionary paths in parallel, up to five variants were selected to become parent sequences for the following round of evolution. To ensure broad exploration of the design space, no positions were modified more than once within a given evolution trajectory. A maximum of one mutation per position was considered for the next round (based on lowest AggScore). A phylogenetic tree connecting selected variants was generated with Geneious Prime,^72,73^ using the Jukes-Cantor genetic distance model and the UPGMA tree build method. The KRED-15 sequence was used as the root for the phylogenetic tree (Figure 2d).

### All-Atom Molecular Dynamics Simulations and Analysis

AAMD simulations were performed using Desmond^74^ within the Schrödinger software package.^75^ Dimeric structures were solvated using an SPC cubic water box, with 15 Å buffer between the protein and the edge of the box. Simulations were conducted under NPT conditions (300 K, 1 bar, 2 fs time step), and Na^+^/Cl^–^ counterions were introduced to neutralize the charge of the system. The Molecular Operating Environment (MOE) software package^76^ was used to identify protein contacts present throughout the AAMD simulations, based on snapshots extracted from the trajectory. Consistent with the contact analysis performed on the CGMD simulations, a cutoff distance of 4 Å was used to identify interchain interactions. MOE was also used to calculate the contact area and interaction energy (GB/VI^77^) at the dimer interface for the sampled protein structures (see Table S6).

## Supplementary Material



## Data Availability

Enzyme structures in this study (all-atom and coarse-grained) were derived from the publicly available 9PHG crystal structure (https://www.rcsb.org/structure/9PHG). The protocol used to run the ViPER workflow for this study is provided in the Supporting Information.

## Notes

Additional information related to structural modeling, molecular dynamics simulations, data analysis, method benchmarking, and the ViPER protocol used in this study (PDF)

## Abbreviations

AAMD: all-atom molecular dynamics
AI: artificial intelligence
CGMD: coarse-grained molecular dynamics
*is*KRED: *in silico* ketoreductase enzyme
KRED: ketoreductase enzyme
KRED-1: monomeric ketoreductase enzyme
KRED-15: engineered, dimeric ketoreductase enzyme
MD: molecular dynamics
ViPER: Virtual Protein EngineeR
