# Supplementary material for: Simulating Directed Evolution In Silico with an Iterative, Physics-Based Workflow
Source: ACS Omega. 2026 Oct 11;11(42):65536–46. doi: 10.1021/acsomega.6c08421 (PMC13717786; doi:10.1021/acsomega.6c08421)
Supplement: Supplementary file 1 [file ao6c08421_si_001.pdf]

Supporting Information

Simulating directed evolution *in silico* with an iterative, physics-based workflow

Chey M. Jones[1*] and Deeptak Verma[1]

[1] Development Sciences and Clinical Supply, Merck & Co., Inc., Rahway, NJ, USA

* Corresponding author

Contents

Abbreviations 2

Section S1: Structural analysis of early-round mutations 3

Section S2: Interaction frequency of KRED residues during CGMD simulations 4

Section S3: Time-resolved separation of *is*KRED dimers during CGMD simulations 5

Section S4: Structural analysis of late-round mutations 6

Section S5: Inter-chain contacts identified during CGMD simulations 7

Section S6: Changes to structural energy and aggregation propensity during *in silico* directed evolution 8

Section S7: Changes to the dimeric interface during *in silico* directed evolution 13

Section S8: Comparison of predicted mutations at the dimer interface – ViPER and generative AI models 14

Section S9: Engineered KRED sites prioritized by ViPER 15

Section S10: Impact of CGMD simulations on the interface contacts identified for *is*KRED structures 16

Section S11: Benchmarking for coarse-grained molecular dynamics simulations 19

Section S12: ViPER protocol 22

References 25

Abbreviations

AAMD – All-atom molecular dynamics

AI – Artificial intelligence

CGMD – Coarse-grained molecular dynamics

*is*KRED – *in silico* ketoreductase enzyme

KRED – ketoreductase enzyme

KRED-1 – monomeric ketoreductase enzyme

KRED-15 – engineered, dimeric ketoreductase enzyme

MD – molecular dynamics

ViPER – Virtual Protein EngineeR

Section S1: Structural analysis of early-round mutations

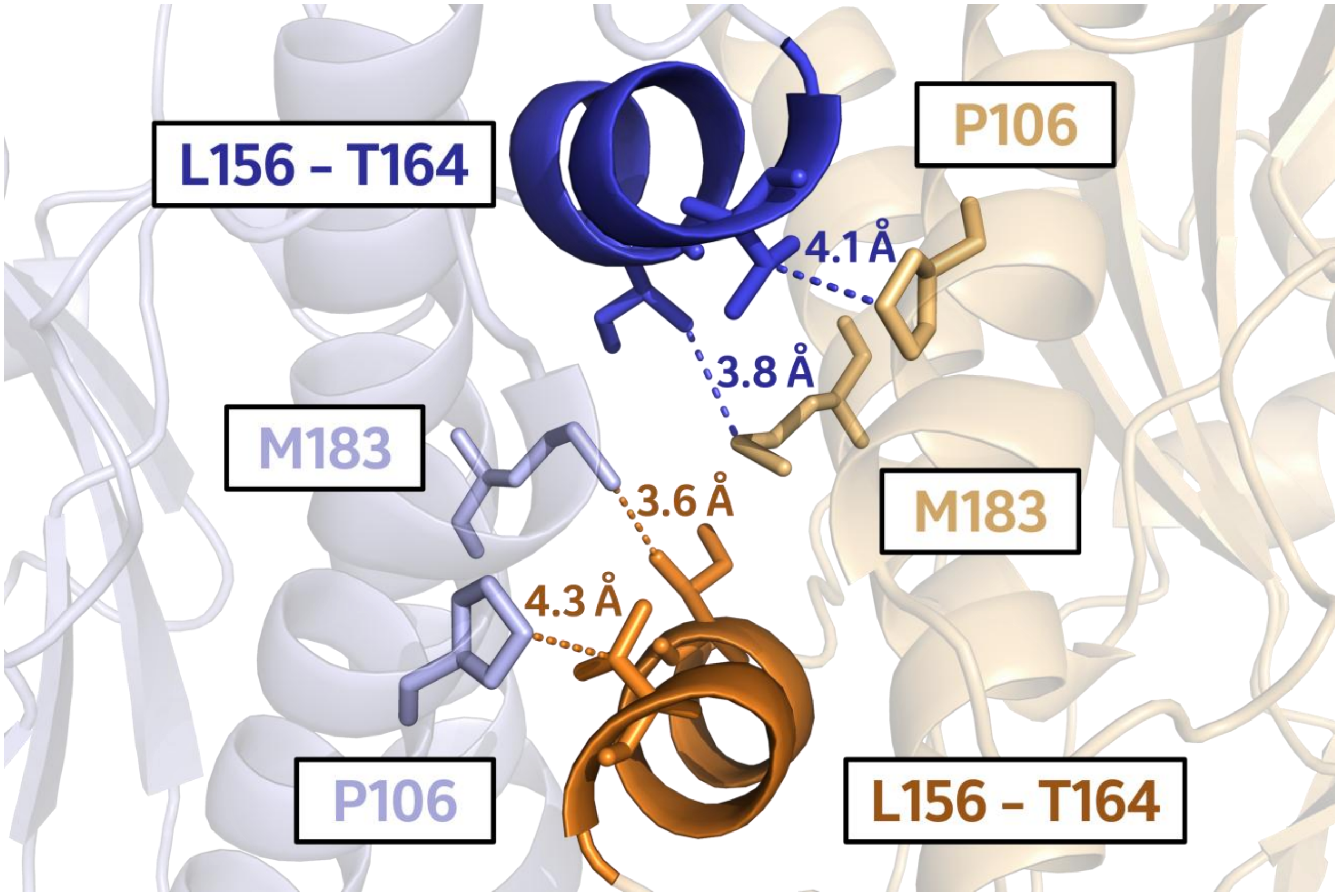

Fig. S1. Location of the P106 and M183 residues that were mutated during Round 1 and Round 2 of *in silico* directed evolution, respectively. Chain A is represented in light blue, highlighting the respective L156-T164 α-helix motif in dark blue. Chain B is represented in light orange, highlighting the respective L156-T164 α-helix motif in dark orange. Distances shown represent the proximity of P106–V163 and I159–M183 in the KRED-15 structure.[1]

Section S2: Interaction frequency of KRED residues during CGMD simulations

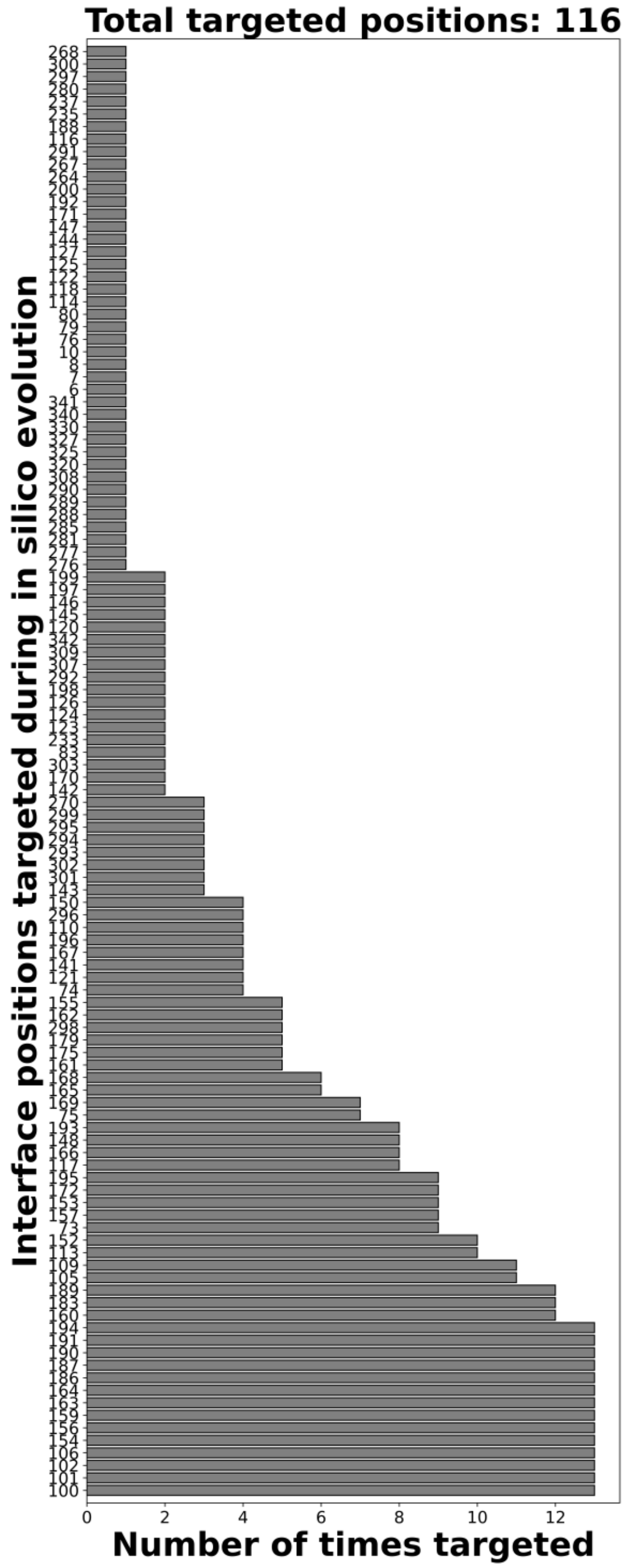

Fig. S2. Number of times that various KRED positions were targeted for *in silico* site saturated mutagenesis after five rounds of *in silico* directed evolution. Each instance represents a unique *is*KRED parent where that position was observed to form a contact with the adjacent *is*KRED chain during a 1 μs CGMD simulation.

Section S3: Time-resolved separation of *is*KRED dimers during CGMD simulations

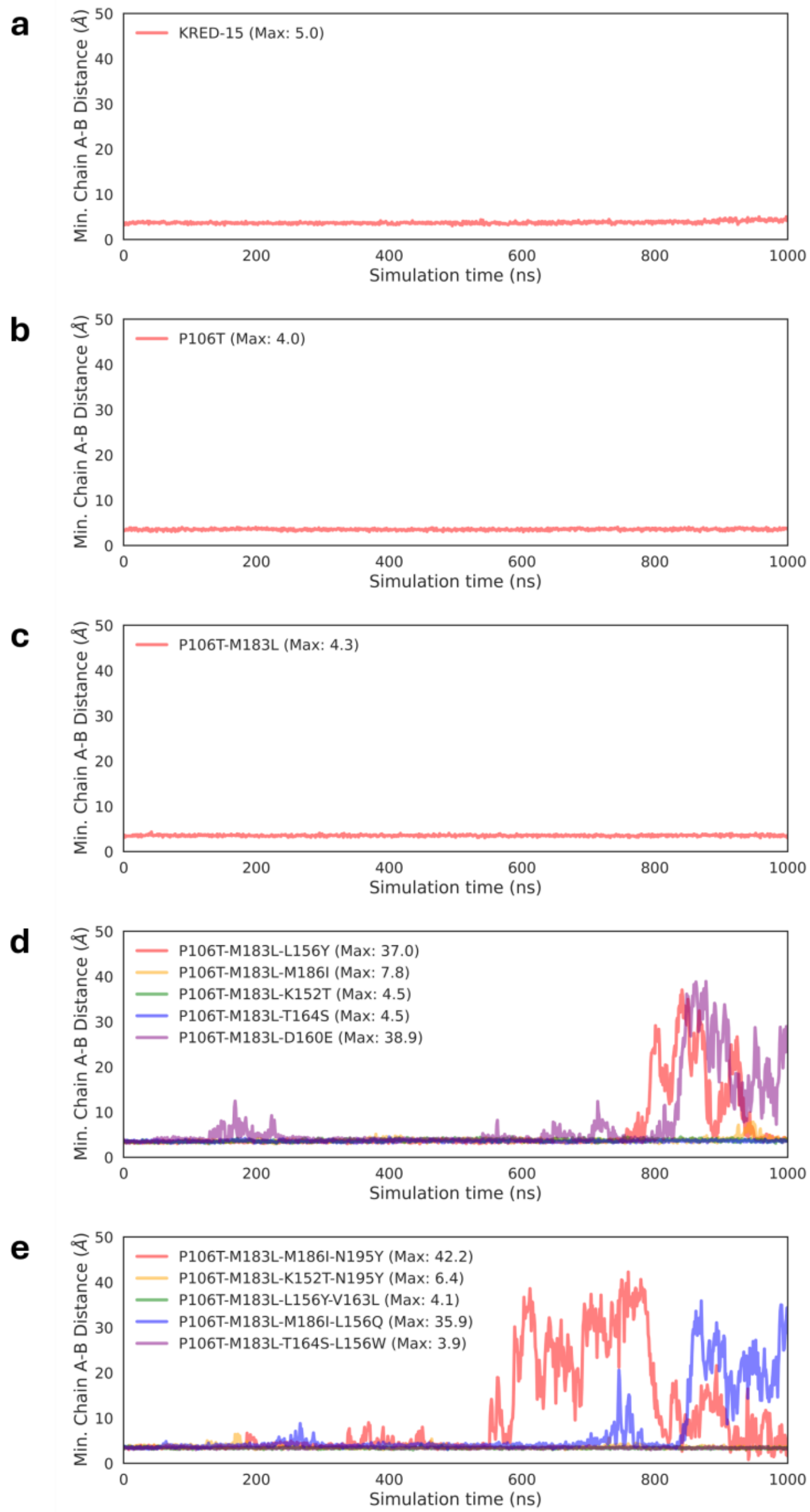

Fig. S3. Minimum distances measured between *is*KRED chains during 1 μs coarse-grained molecular dynamics simulations. Measured values for the *is*KRED parents used in Rounds 1-5 of *in silico* directed evolution are represented in subfigures **a-e**, respectively. Values in parenthesis represent the largest separation measured between the chains during the simulation.

Section S4: Structural analysis of late-round mutations

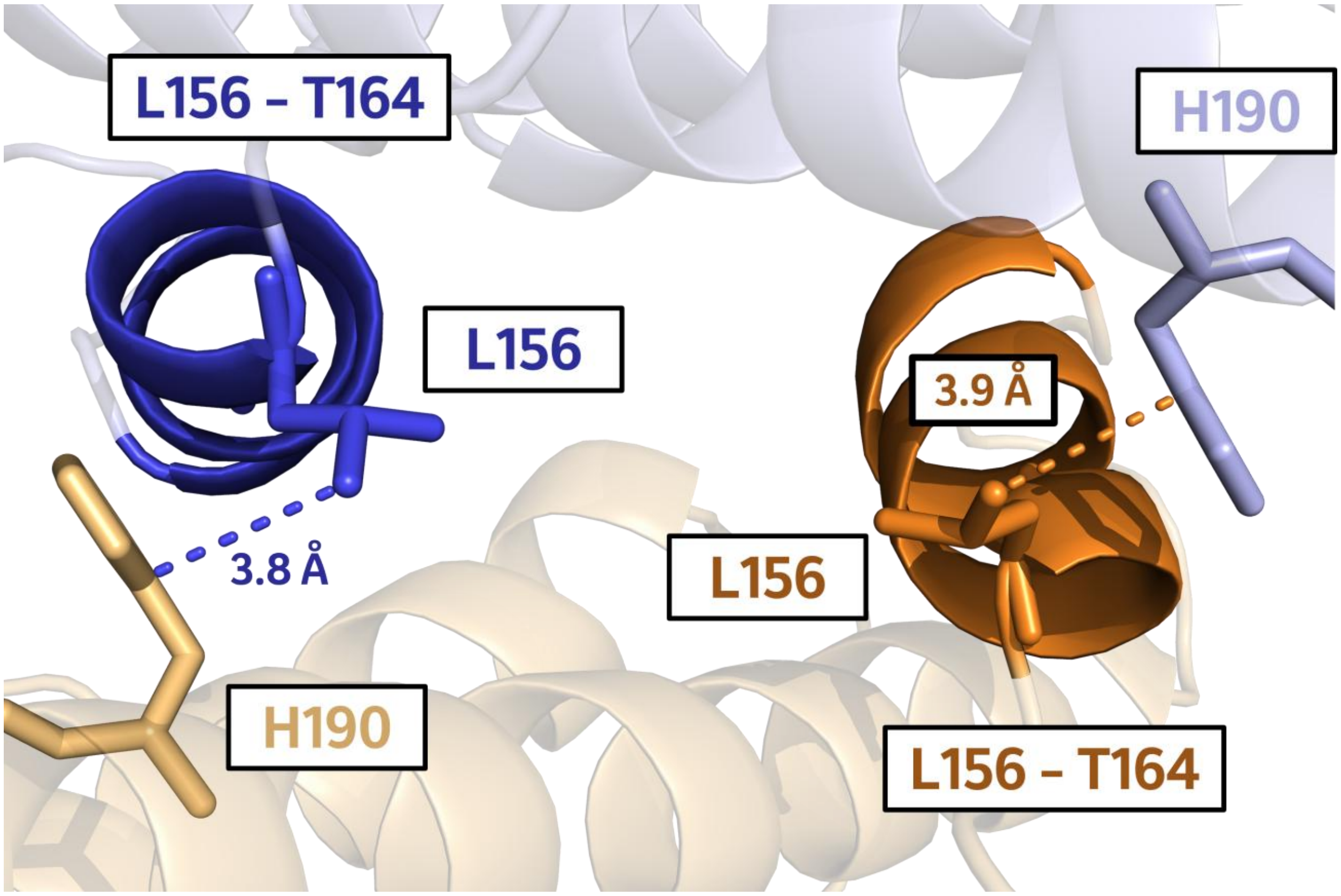

Fig. S4. Location of the L156 residue that was mutated during Round 4 of *in silico* directed evolution. Chain A is represented in light blue, highlighting the respective L156-T164 α-helix motif in dark blue. Chain B is represented in light orange, highlighting the respective L156-T164 α-helix motif in dark orange. Distances shown represent the proximity of L156–H190 in the parent KRED-15 structure.

Section S5: Inter-chain contacts identified during CGMD simulations

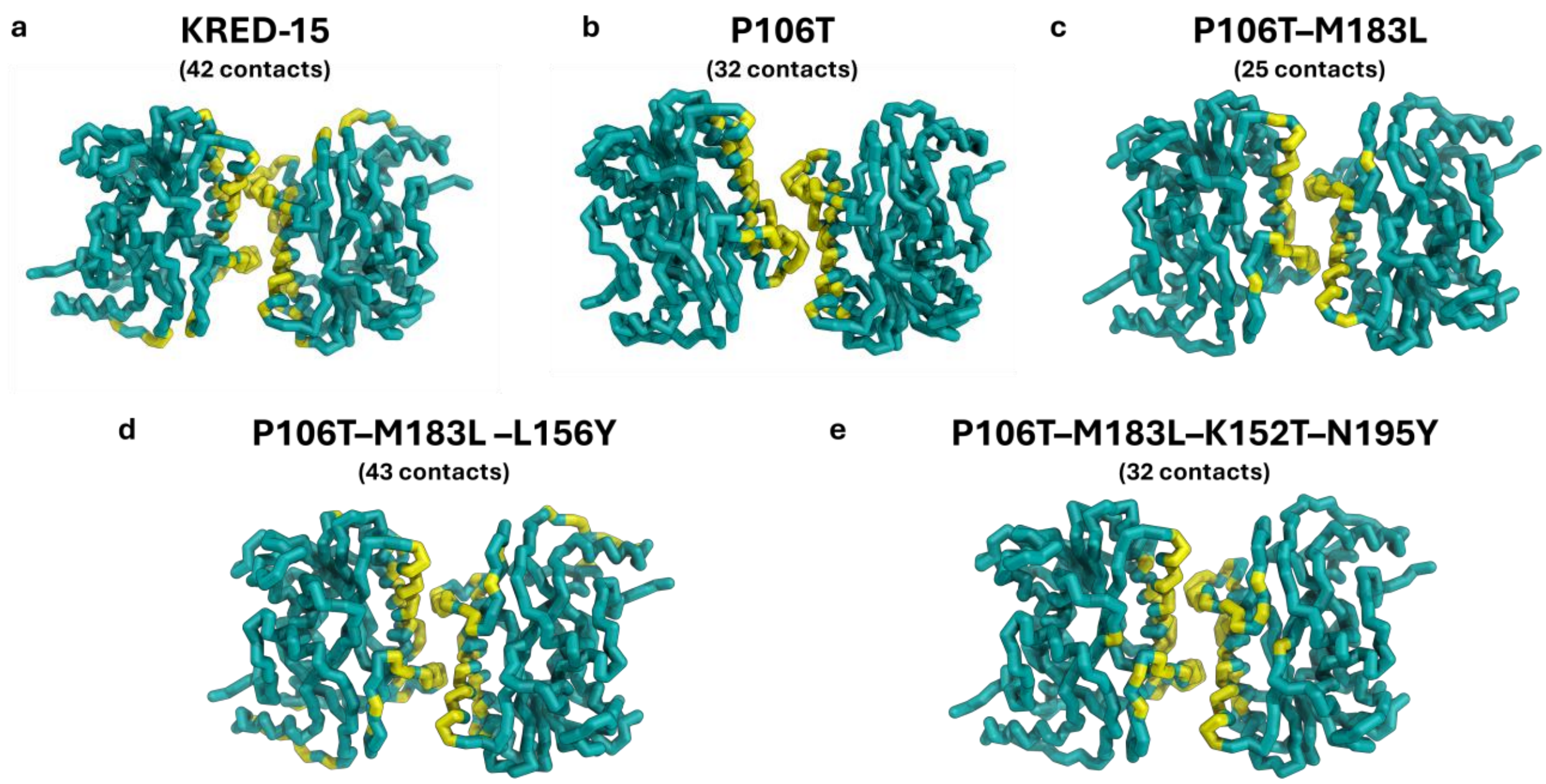

Fig. S5. Inter-chain contacts (within 4 Å) identified during coarse-grained molecular dynamics simulations of parent *is*KRED enzymes. **a-e** Parent *is*KRED enzymes with the lowest predicted dimer AggScore are shown for Rounds 1-5, respectively. Residues that form contacts with the adjacent chain are highlighted in yellow.

Section S6: Changes to structural energy and aggregation propensity during *in silico* directed evolution

Table S1. Calculated ΔΔG and AggScore values for the top five variants evaluated in Round 1 of *in silico* directed evolution. The top variants were identified based on the calculated AggScore of their dimeric structure, prioritizing variants with low dimer AggScores. AggScores for the monomers of each variant are tabulated as well. ΔΔG values for the monomeric and dimeric forms of the enzyme variants were calculated with Rosetta, using the energy of the Round 1 Parent enzyme as a reference. Variants chosen as parents for the next round are highlighted using an asterisk.

***is*KRED Variant**	**Monomer ΔΔG, rel. to Round 1 Parent (REU)**	**Dimer ΔΔG, rel. to Round 1 Parent (REU)**	**Monomer AggScore**	**Dimer AggScore**
**P106T***	-4.6	-35.8	323	503
P106K	-11.3	-37.0	309	507
P106R	-7.0	-31.4	297	510
P106I	-6.8	-34.8	342	511
P106M	-8.4	-35.6	347	517

After the first round of *in silico* evolution, there were thirteen energetically favorable options to initiate our computational engineering workflow. All of these mutation suggestions were at position 106: P106T, P106K, P106R, P106I, P106M, P106Q, P106A, P106G, P106F, P106S, P106V, P106L, and P106W. Although these thirteen mutations were predicted to be energetically accessible, they all had predicted AggScores that were greater than that of the KRED-15 parent enzyme (*i.e.,* 493). To avoid choosing mutations that increased the AggScore too drastically, the P106T variant was selected as the first mutation to begin our *in silico* directed evolution campaign – since it has the lowest predicted AggScore across all P106 variants in Round 1.

Table S2. Calculated ΔΔG and AggScore values for the top five variants evaluated in Round 2 of *in silico* directed evolution. The top variants were identified based on the calculated AggScore of their dimeric structure, prioritizing variants with low dimer AggScores. Mutations at positions that were modified in previous rounds were excluded from consideration. AggScores for the monomers of each variant are tabulated as well. ΔΔG values for the monomeric and dimeric forms of the enzyme variants were calculated with Rosetta, using the energy of the Round 2 Parent enzyme as a reference. Variants chosen as parents for the next round are highlighted using an asterisk.

***is*KRED Variant**	**Monomer ΔΔG, rel. to Round 2 Parent (REU)**	**Dimer ΔΔG, rel. to Round 2 Parent (REU)**	**Monomer AggScore**	**Dimer AggScore**
**P106T-M183L***	-2.3	-8.1	312	480
P106T–I159M†	-0.5	-9.2	310	469
P106T–L73R†	-1.2	-7.7	288	486
P106T–K161A†	-0.3	-2.1	309	506
P106T–E194V†	-0.5	-6.1	324	507

† These mutations were de-prioritized since their calculated monomer/dimer ΔΔG values were within the standard deviation of the ΔG values calculated for the *is*KRED Round 2 parent enzyme.

Table S3. Calculated ΔΔG and AggScore values for the top five variants evaluated in Round 3 of *in silico* directed evolution. The top variants were identified based on the calculated AggScore of their dimeric structure, prioritizing variants with low dimer AggScores. Mutations at positions that were modified in previous rounds were excluded from consideration. AggScores for the monomers of each variant are tabulated as well. ΔΔG values for the monomeric and dimeric forms of the enzyme variants were calculated with Rosetta, using the energy of the Round 3 Parent enzyme as a reference. Variants chosen as parents for the next round are highlighted using an asterisk.

***is*KRED Variant**	**Monomer ΔΔG, rel. to Round 3 Parent (REU)**	**Dimer ΔΔG, rel. to Round 3 Parent (REU)**	**Monomer AggScore**	**Dimer AggScore**
**P106T-M183L-L156Y***	-4.7	-6.5	299	443
**P106T-M183L-T164S***	-6.6	-5.5	285	483
**P106T-M183L-K152T***	-3.9	-12.0	312	493
**P106T-M183L-M186I***	-3.5	-8.5	316	495
**P106T-M183L-D160E***	-3.9	-15.7	309	497

Table S4. Calculated ΔΔG and AggScore values for the top five variants evaluated in Round 4 of *in silico* directed evolution. The top variants were identified based on the calculated AggScore of their dimeric structure, prioritizing variants with low dimer AggScores. Mutations at positions that were modified in previous rounds were excluded from consideration. AggScores for the monomers of each variant are tabulated as well. ΔΔG values for the monomeric and dimeric forms of the enzyme variants were calculated with Rosetta, using the energy of the Round 4 Parent enzyme as a reference. Variants chosen as parents for the next round are highlighted using an asterisk.

***is*KRED Variant**	**Monomer ΔΔG, rel. to Round 4 Parent (REU)**	**Dimer ΔΔG, rel. to Round 4 Parent (REU)**	**Monomer AggScore**	**Dimer AggScore**
**P106T-M183L-K152T-N195Y***	-5.9	-8.4	318	452
**P106T-M183L-T164S-L156W***	-3.7	-6.5	323	460
**P106T-M183L-M186I-L156Q***	-3.7	-4.6	337	462
**P106T-M183L-L156Y-V163L***	-2.4	-8.9	321	464
**P106T-M183L-M186I-N195Y***	-2.6	-6.9	317	470

Table S5. Calculated ΔΔG and AggScore values for the top five variants evaluated in Round 5 of *in silico* directed evolution. The top variants were identified based on the calculated AggScore of their dimeric structure, prioritizing variants will low dimer AggScores. Mutations at positions that were modified in previous rounds were excluded from consideration. AggScores for the monomers of each variant are tabulated as well. ΔΔG values for the monomeric and dimeric forms of the enzyme variants were calculated with Rosetta, using the energy of the Round 5 Parent enzyme as a reference. Variants chosen as parents for the next round are highlighted using an asterisk.

***is*KRED Variant**	**Monomer ΔΔG, rel. to Round 5 Parent (REU)**	**Dimer ΔΔG, rel. to Round 5 Parent (REU)**	**Monomer AggScore**	**Dimer AggScore**
**P106T-M183L-M186I-N195Y-W291Y***	-9.7	-6.9	320	445
**P106T-M183L-M186I-L156Q-V235N***	-3.1	-3.8	337	446
**P106T-M183L-M186I-L156Q-N195Y***	-2.6	-4.1	344	450
**P106T-M183L-M186I-N195Y-I159M***	-6.3	-4.7	333	453
**P106T-M183L-M186I-N195Y-A121K***	-9.8	-3.7	344	454

Section S7: Changes to the dimeric interface during *in silico* directed evolution

Table S6. Interface interaction energy and interface contact area calculated for KRED-1, KRED-15, and *is*KRED parents obtained during *in silico* directed evolution. Averages and standard deviations for each KRED variant were calculated using structures obtained during the final 100 ns of their respective 200 ns all-atom molecular dynamics simulation. In total, 21 evenly spaced snapshots were used to calculate the statistics for this analysis.

Variant	Interface interaction energy, GB/VI (kcal/mol)	Interface contact area (Å^2)
KRED-15	-72.9 ± 5.1	1276 ± 51
P106T	-66.7 ± 4.6	1278 ± 53
P106T–M183L	-63.2 ± 5.4	1218 ± 43
P106T–M183L– L156Y	-66.6 ± 4.1	1280 ± 56
P106T–M183L–K152T–N195Y	-60.0 ± 3.3	1236 ± 49
P106T–M183L–M186I–N195Y–W291Y	-71.9 ± 6.0	1346 ± 60
KRED-1	-44.2 ± 5.1	754 ± 33

Section S8: Comparison of predicted mutations at the dimer interface – ViPER and generative AI models

To evaluate ViPER's ability to target the key sites hypothesized to toggle KRED oligomerization versus alternative methods, we obtained predicted mutations suggested by various generative AI models. Sequence-based predictions were generated using ESM[2,3] (1v and inverse folding, IF, models), and structure-based predictions were generated using ProteinMPNN[4] and SolubleMPNN.[5] Based on this comparative analysis, it appears that the dynamic and energetic filtering methodology employed by ViPER efficiently narrows the design space while suggesting mutations at the crowded protein-protein interface. Relative to the four zero-shot methods, ViPER identified the most sites responsible for toggling KRED oligomerization. However, there does appear to be complementarity between the physics-based approach of ViPER and these generative AI methods. For example, we observe that the ESM-1v model predicts viable mutagenesis routes involving position 187, which is an interfacial patch position that was not captured during the first five rounds of *in silico* evolution with ViPER.

Table S7. Performance comparison of *in silico* prediction methods, based on their ability to target interfacial positions hypothesized to toggle KRED oligomerization (*i.e.,* positions 94, 106, 163, 183, 186, 187, 190). Predictions from the ViPER workflow were obtained from data gathered during five rounds of *in silico* directed evolution. Predictions from various generative AI methods were obtained based on zero-shot predictions using the sequence and structure of the KRED-15 dimer (PDB ID: 9PHG[1]). The predicted number of viable mutations for each generative AI method was determined based on the model's scoring preference toward a mutated residue, relative its original residue.

Prediction method	Number of engineerable sites suggested	Number of suggested mutations	Engineered interface patch residues identified	Top mutation predictions at engineered interface patch residues
ViPER	28	156	5 / 7	P106T, V163L, M183L, M186I, H190W
ESM-1v	11	18	3 / 7	M186K, L187A, H190K
ESM-IF	78	232	4 / 7	V163T, M183I, M186K, H190K
ProteinMPNN	107	295	2 / 7	V163F, M186L
SolubleMPNN	115	305	0 / 7	---

Section S9: Engineered KRED sites prioritized by ViPER

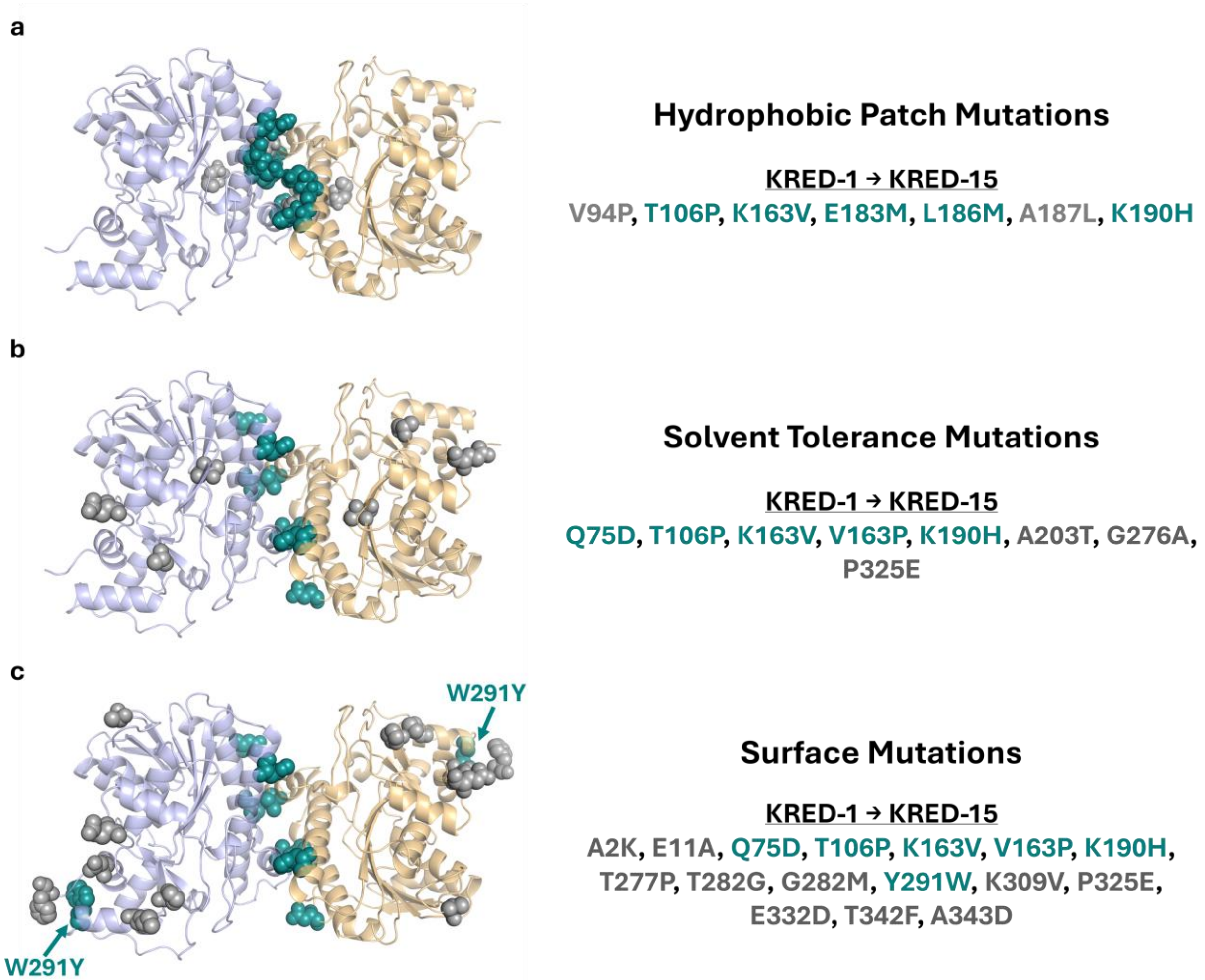

Fig. S6. Summary of the hydrophobic patch (**a**), solvent tolerance (**b**) and surface mutations (**c**) accumulated during the experiment-based engineering of KRED-15 (shown as spheres).[1] Positions highlighted in teal represent positions identified as high-priority sites by ViPER. Positions highlighted in gray represent positions de-prioritized by ViPER.

Section S10: Impact of CGMD simulations on the interface contacts identified for *is*KRED structures

Table S8. Number of residues that form interface contacts (*i.e.,* within 4 Å) for five parent *is*KRED enzymes selected during *in silico* evolution. Static structures were generated *via* homology modeling, and a 4 Å cutoff was used to identify interface contacts between the two chains of the *is*KRED dimer. CGMD simulation contacts (within 4 Å) were obtained from 1 μs simulations for each of the specified *is*KRED parent enzymes.

Variant	Residues that form interface contacts, Static structures*	Residues that form interface contacts, CGMD simulation
KRED-15	17	42
P106T	17	32
P106T–M183L	17	25
P106T–M183L– L156Y	17	43
P106T–M183L–K152T–N195Y	17	35
**Total unique positions explored**	**17**	**116**

***** The 17 interface positions obtained from static-structure analysis include: 101, 102, 106, 109, 110, 113, 117, 156, 157, 159, 160, 163, 164, 183, 186, 187, 190.

Table S9. List of mutations reported by Chun *et al.* to engineer the KRED-15 dimer from the KRED-1 dimer.[1] To assess the value of integrating CGMD simulations into the ViPER workflow, each mutated position was classified according to whether or not it was predicted to be an important interface position from either (a) analysis of a static structure or (b) analysis of CGMD simulations obtained across five rounds of *in silico* directed evolution. An asterisk indicates that an energetically accessible mutation was predicted by ViPER at that position (after five rounds of *in silico* evolution).

**KRED-1 to KRED-15 Mutation**	**Position identified by static structure analysis?**	**Position identified by CGMD simulation analysis?**
K2A	No	No
E11A	No	No
Q75D	No	Yes*
V94P	No	No
S96L; L96S	No	No
S98Q; Q98S	No	No
N99P	No	No
T106P	Yes*	Yes*
T134M	No	No
V135M	No	No
S136A	No	No
L138Y; Y138I	No	No
I139V	No	No
E145Q; Q145P	No	Yes
K163V	Yes*	Yes*
D169H	No	Yes
P170K	No	Yes
Q171Y; Y171E	No	Yes
S173G	No	No
E183T; T183M	Yes*	Yes*
L186M	Yes*	Yes*
A187L	Yes	Yes
K190H	Yes*	Yes*
A203T	No	No
L205A; A205L	No	No
N207A	No	No

W226F; F226I	No	No
M227V	No	No
S229K	No	No
A238M	No	No
M242F; F242V; V242F	No	No
P243R; R243G	No	No
Y246H	No	No
V251F	No	No
G276A	No	Yes
T277P	No	Yes
T282M	No	No
T286V	No	No
Y291W	No	Yes*
K309V	No	Yes
A313R	No	No
P325E	No	Yes
I330F	No	Yes
E332D	No	No
T342F	No	Yes
A343D	No	No
**Engineered positions captured**	**6 out of 46 (13%)**	**18 out of 46 (39%)**
**Stable mutation predicted at engineered position**	**5 out of 46 (10%)**	**7 out of 46 (15%)**

Section S11: Benchmarking for coarse-grained molecular dynamics simulations

Parameter selection. Parameters for the generation of coarse-grained, dimeric KRED structures were obtained based on consistency with all-atom molecular dynamics simulations. Various coarse-grained parameters (*e.g.,* elastic bond force constant, elastic bond cutoffs) were used to initialize coarse-grained molecular dynamics simulations, and the final parameters were chosen based on their ability to mimic the short- and long-term behavior observed during all-atom molecular dynamics simulations. The KRED-15 dimer was used for this benchmarking procedure. Based on this benchmarking, lower- and upper-bound elastic bond cutoffs were defined as 0.5 nm and 0.9 nm, respectively. The harmonic elastic bond force constant was set to 700 kJ/(mol·nm^2).

To test the robustness of the coarse-grained parameters chosen, we ran triplicate 1 μs CGMD simulations to compare to our 1 μs AAMD simulation reference. The AAMD simulations generally had RMSD values that fluctuated between 1.9 Å and 2.5 Å for KRED-15 chains A and B. To demonstrate consistency across multiple CGMD simulations, we calculated the RMSD of both chains during all three triplicate CGMD runs. Across these three KRED-15 trajectories, we observe RMSD values that generally fluctuate between 1.6 Å and 2.0 Å – which overlaps with the desired range established by our AAMD reference (**Fig. S7**).

CGMD replicate simulations. We identified variability in the number of interface contacts across CGMD replicate simulations (**Table S10**). Considering ViPER was designed to enable a physics-based exploration of enzyme design space, it is promising to observe that starting simulations with different initial conditions can lead to the sampling of unique evolutionary pathways. Relative to the rigid set of positions obtained from static structures (see **Table S8**), which highly constrains any exploration efforts, our CGMD workflow identified 116 possible positions to explore for this *in silico* evolution.

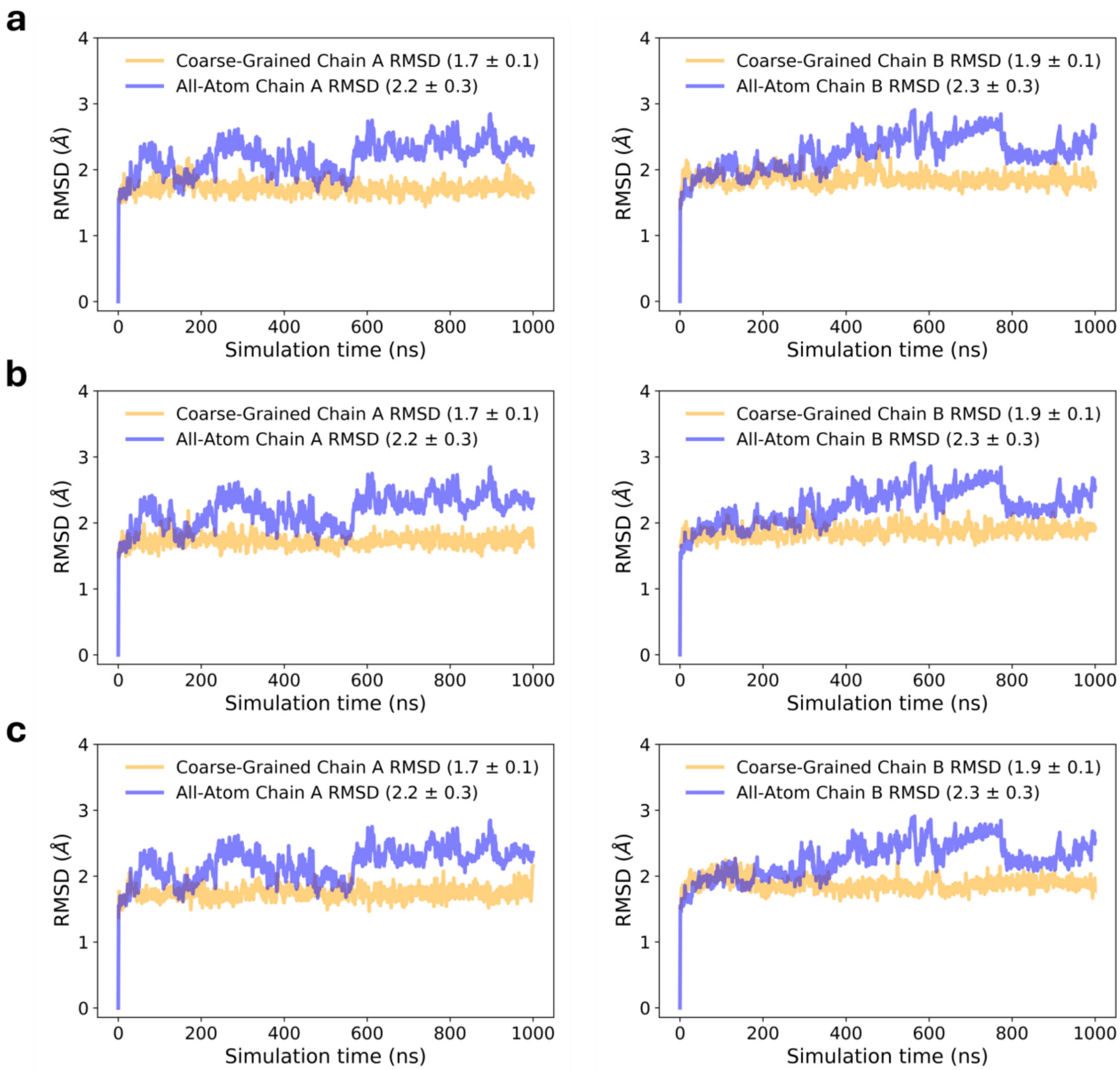

Fig. S7. Root mean square deviation (RMSD) values calculated for the KRED-15 dimer chains during 1 μs of coarse-grained molecular dynamics simulations (orange) and an all-atom molecular dynamics simulation (blue). Subplots **a**, **b**, and **c** correspond to three replicates of the coarse-grained molecular dynamics simulation. In each subplot, the same all-atom molecular dynamics simulation is used as a reference. Values in parentheses represent calculated averages and standard deviations for their respective trajectory.

Table S10. Number of residues that form interfacial contacts throughout each of the triplicate CGMD simulations of KRED-15 used for benchmarking. Each CGMD simulation started from the same initial structure and ran for 1 μs.

KRED-15 CGMD Replicate	Residues that form interface interactions	Interacting residues that overlap with Trajectory 1	Interacting residues that overlap with Trajectory 2	Interacting residues that overlap with Trajectory 3
Trajectory 1	42	---	21	29
Trajectory 2	33	21	---	19
Trajectory 3	31	29	19	---

Section S12: ViPER protocol

Step 1: Obtain protein sequences and structures for the parent enzymes

For this study, the sequence and structure of a dimeric ketoreductase of interest (9PHG[1]) were obtained from the publicly accessible PDB database: https://www.rcsb.org/structure/9PHG

Step 2: Generate atomistic models of the parent enzymes

All-atom structures for the parent enzymes were generated using Schrödinger's Maestro suite.[6] The dimeric, 9PHG structure was used as the structural template for all *is*KRED variants in this study. The Methods section in the Main Text provides the relevant details for the atomistic modeling protocol used in this study.

Step 3: Generate solvated coarse-grained structures of the parent enzymes

Coarse-grained structures for the parent enzymes were generated using the publicly available martinize2 protocol,[7-9] available on GitHub (https://github.com/marrink-lab/vermouth-martinize). Secondary structure information (DSSP) was obtained using the publicly available mdtraj python package, available on GitHub (https://github.com/mdtraj/mdtraj).[9] These coarse-grained structures were solvated using the insane protocol,[10] which is publicly available on GitHub (https://github.com/Tsjerk/Insane). When constructing the elastic-network representation of the enzyme, the default martinize2 protocol was used, such that elastic bonds were generated only within individual protein monomers and not between monomers. Martini3[7,11-15] was used to parameterize the coarse-grained models, and the details for this coarse-graining procedure are provided in the Methods section of the Main Text. The command-line methodology is provided below:

Coarse-grained structure generation: *martinize2 -f 9PHG.pdb -x dimer_cg.pdb -o dimer_only.top -ff martini3001 -p backbone -dssp -elastic -ef 700 -el 0.5 -eu 0.9*

Solvated system generation: *python2.7 insane.py -f dimer_cg.pdb -o cg_system.gro -p cg_system.top -pbc cubic -box 15,15,15 -salt 0.15 -sol W -d 1.0 -charge auto*

Step 4: Coarse-grained molecular dynamics simulations

The output files associated with the solvated protein systems were formatted to ensure compatibility with the Martini3 force field and GROMACS.[16-21] Subsequently, the solvated

systems were subjected to minimization, equilibration, and production-scale molecular dynamics simulations. Details regarding the simulation parameters are provided in the Methods section of the Main Text. The command-line methodology is provided below:

Minimization:

gmx_mpi grompp -p cg_system.top -c cg_system.gro -f minimization.mdp -o minimization.tpr -r cg_system.gro

gmx_mpi mdrun -v -deffnm minimization -ntomp 6

Equilibration:

gmx_mpi grompp -p cg_system.top -c minimization.gro -f equilibration.mdp -o equilibration.tpr -r minimization.gro -maxwarn 3

gmx_mpi mdrun -v -deffnm equilibration -ntomp 6

Production:

gmx_mpi grompp -p cg_system.top -c equilibration.gro -f md.mdp -o production.tpr

gmx_mpi mdrun -deffnm production -ntomp 6

Step 5: Trajectory post-processing and trajectory analysis

To enable consistent analysis across multiple simulations, the trajectory of each simulation was centered and aligned using GROMACS functions that enabled the most consistent alignment of the *is*KRED dimers:

Center protein atoms in the PBC box: *gmx_mpi trjconv -s production.tpr -f production.xtc -center -pbc nojump -o md_center.xtc*

Fit trajectory to backbone atoms in the first frame: *gmx_mpi trjconv -s production.tpr -f md_center.xtc -fit rot+trans -o md_fit.xtc*

The post-processed trajectories of the coarse-grained systems were then analyzed using standard GROMACS functions to obtain structural information associated with the molecular dynamics simulations:

RMSD analysis: *gmx_mpi rms -s production.tpr -f md_fit.xtc -o rmsd*

RMSF analysis: *gmx_mpi rmsf -s production.tpr -f md_fit.xtc -o rmsf*

SASA analysis: *gmx_mpi sasa -s production.tpr -f md_fit.xtc -or -oa*

These aligned trajectories were then used to identify distance-based interactions at the KRED's dimeric interface. This analysis was performed using mdtraj,[9] and the details of this analysis are provided in the Methods section of the Main Text.

Step 6: *in silico* site-saturated mutagenesis with Rosetta

Rosetta software[22-24] was used to perform *in silico* site-saturated mutagenesis at the interaction sites identified in the previous step. These Rosetta calculations used the atomistic homology models, associated with their respective parents, as input. The details of this methodology are provided in the Methods section of the Main Text.

Step 7: Structure-based variant evaluation and selection

Schrödinger software[6] was used to calculate protein descriptors on the Rosetta-generated *is*KRED variant structures. Protein descriptors were calculated at a pH of 7.4:

Structure-based variant analysis: *$SCHRODINGER/run -FROM psp calc_protein_descriptors.py -i stable_dimer_variants -o stable_dimer_variants_descriptors --pH 7.4*

The top variants within a given round were selected based on calculated AggScore,[25] as described in the Methods section of the Main Text.

References

1 Chun, S. W. *et al.* Ketoreductase Engineering for a Chemoenzymatic Fluorination and Dynamic Kinetic Reduction Cascade. *ACS Catalysis* **15**, 13059-13068 (2025). https://doi.org/10.1021/acscatal.5c01769

2 Lin, Z. *et al.* Evolutionary-scale prediction of atomic-level protein structure with a language model. *Science* **379**, 1123-1130 (2023). https://doi.org/10.1126/science.ade2574

3 Hayes, T. *et al.* Simulating 500 million years of evolution with a language model. *Science* **387**, 850-858 (2025). https://doi.org/10.1126/science.ads0018

4 Dauparas, J. *et al.* Robust deep learning-based protein sequence design using ProteinMPNN. *Science* **378**, 49-56 (2022). https://doi.org/10.1126/science.add2187

5 Goverde, C. A. *et al.* Computational design of soluble and functional membrane protein analogues. *Nature* **631**, 449-458 (2024). https://doi.org/10.1038/s41586-024-07601-y

6 Schrödinger Release 2025-2: Schrödinger, LLC, New York, NY, 2025. (2025).

7 Souza, P. C. T. *et al.* Martini 3: a general purpose force field for coarse-grained molecular dynamics. *Nat Methods* **18**, 382-388 (2021). https://doi.org/10.1038/s41592-021-01098-3

8 Kroon, P. C. *et al.* Martinize2 and Vermouth: Unified Framework for Topology Generation. *eLife* **12** (2023). https://doi.org/10.7554/eLife.90627.2

9 McGibbon, R. T. *et al.* MDTraj: A Modern Open Library for the Analysis of Molecular Dynamics Trajectories. *Biophys J* **109**, 1528-1532 (2015). https://doi.org/10.1016/j.bpj.2015.08.015

10 Wassenaar, T. A., Ingolfsson, H. I., Bockmann, R. A., Tieleman, D. P. & Marrink, S. J. Computational Lipidomics with insane: A Versatile Tool for Generating Custom Membranes for Molecular Simulations. *J Chem Theory Comput* **11**, 2144-2155 (2015). https://doi.org/10.1021/acs.jctc.5b00209

11 Periole, X. & Marrink, S. J. The Martini coarse-grained force field. *Methods Mol Biol* **924**, 533-565 (2013). https://doi.org/10.1007/978-1-62703-017-5_20

12 de Jong, D. H. *et al.* Improved Parameters for the Martini Coarse-Grained Protein Force Field. *J Chem Theory Comput* **9**, 687-697 (2013). https://doi.org/10.1021/ct300646g

13 Risselada, H. J. Martini 3: a coarse-grained force field with an eye for atomic detail. *Nat Methods* **18**, 342-343 (2021). https://doi.org/10.1038/s41592-021-01111-9

14 Alessandri, R. *et al.* Martini 3 Coarse-Grained Force Field: Small Molecules. *Advanced Theory and Simulations* **5** (2021). https://doi.org/10.1002/adts.202100391

15 Marrink, S. J. *et al.* Two decades of Martini: Better beads, broader scope. *WIREs Computational Molecular Science* **13** (2022). https://doi.org/10.1002/wcms.1620

16 Abraham, M. J. *et al.* GROMACS: High performance molecular simulations through multi-level parallelism from laptops to supercomputers. *SoftwareX* **1-2**, 19-25 (2015). https://doi.org/10.1016/j.softx.2015.06.001

17 Pronk, S. *et al.* GROMACS 4.5: a high-throughput and highly parallel open source molecular simulation toolkit. *Bioinformatics* **29**, 845-854 (2013). https://doi.org/10.1093/bioinformatics/btt055

18 Hess, B., Kutzner, C., van der Spoel, D. & Lindahl, E. GROMACS 4: Algorithms for Highly Efficient, Load-Balanced, and Scalable Molecular Simulation. *J Chem Theory Comput* **4**, 435-447 (2008). https://doi.org/10.1021/ct700301q

19 Van Der Spoel, D. *et al.* GROMACS: fast, flexible, and free. *J Comput Chem* **26**, 1701-1718 (2005). https://doi.org/10.1002/jcc.20291

20 Lindahl, E., Hess, B. & van der Spoel, D. GROMACS 3.0: a package for molecular simulation and trajectory analysis. *Journal of Molecular Modeling* **7**, 306-317 (2001). https://doi.org/10.1007/s008940100045

21 Berendsen, H. J. C., van der Spoel, D. & van Drunen, R. GROMACS: A message-passing parallel molecular dynamics implementation. *Computer Physics Communications* **91**, 43-56 (1995). https://doi.org/10.1016/0010-4655(95)00042-e

22 Rohl, C. A., Strauss, C. E., Misura, K. M. & Baker, D. Protein structure prediction using Rosetta. *Methods Enzymol* **383**, 66-93 (2004). https://doi.org/10.1016/S0076-6879(04)83004-0

23 Leaver-Fay, A. *et al.* ROSETTA3: an object-oriented software suite for the simulation and design of macromolecules. *Methods Enzymol* **487**, 545-574 (2011). https://doi.org/10.1016/B978-0-12-381270-4.00019-6

24 Leman, J. K. *et al.* Macromolecular modeling and design in Rosetta: recent methods and frameworks. *Nat Methods* **17**, 665-680 (2020). https://doi.org/10.1038/s41592-020-0848-2

25 Sankar, K., Krystek, S. R., Jr., Carl, S. M., Day, T. & Maier, J. K. X. AggScore: Prediction of aggregation-prone regions in proteins based on the distribution of surface patches. *Proteins* **86**, 1147-1156 (2018). https://doi.org/10.1002/prot.25594
